# Genome-Wide Comparative Analysis of the Fasciclin-like Arabinogalactan Proteins (FLAs) in *Salicacea* and Identification of Secondary Tissue Development-Related Genes

**DOI:** 10.3390/ijms24021481

**Published:** 2023-01-12

**Authors:** Yingying Zhang, Fangwei Zhou, Hui Wang, Yingnan Chen, Tongming Yin, Huaitong Wu

**Affiliations:** Key Laboratory for Tree Breeding and Germplasm Improvement, Southern Modern Forestry Collaborative Innovation Center, College of Forestry, Nanjing Forestry University, Nanjing 210037, China

**Keywords:** *Populus deltoides*, *Salix suchowensis*, fasciclin-like arabinogalactan proteins, gene family, secondary growth, seed hair

## Abstract

Fasciclin-like arabinogalactan proteins (FLAs) are a subclass of arabinogalactan proteins (AGPs) containing both AGP-like glycated domains and fasciclin (FAS) domains, which are involved in plant growth and development and synthesis of the cell wall. However, these proteins have not been identified or analyzed in willow, *Salix*, the sister genus of *Populus*. In this study, we performed a whole genome study of the FLA gene family of *Salix suchowensis* and compared it with the FLA gene family of *Populus deltoides*. The results showed the presence of 40 and 46 FLA genes in *P. deltoides* and *S. suchowensis*, distributed on 17 and 16 chromosomes, respectively. Four pairs of tandem repeat genes were found in willow, while poplar had no tandem repeat genes. Twelve and thirteen pairs of duplicated gene fragments were identified in poplar and willow, respectively. The multispecies phylogenetic tree showed that the FLA gene family could be divided into four groups (I–IV), with Group 1 showing significant expansion in woody plants. A gene expression analysis showed that *PdeFLA19/27* in Group I of poplar was highly expressed, specifically during the secondary growth period of the stem and the rapid elongation of seed hairs. In the Group I genes of *S. suchowensis*, *SsuFLA25/26/28* was also highly expressed during the secondary growth period, whereas increased expression of *SsuFLA35* was associated with seed hair tissue. These results provide important clues about the differences in the FLA gene family during the evolution of herbs and woody plants, and suggest that the FLA gene family may play an essential role in regulating the secondary growth of woody plants. It also provides a reference for further studies on the regulation of secondary growth and seed hair development by FLA genes in poplar and willow.

## 1. Introduction

The hydroxyproline-rich glycoproteins (HRGPs) are a class of structural proteins usually found in the cell walls of higher plants [1,2,3,4]. Arabinogalactan proteins (AGPs) are a subfamily of HRGPs involved in many aspects of plant development, including tissue differentiation, reproduction, plant hormone interactions, responses to biotic stress, cell expansion, and secondary cell wall deposition [5,6]. AGPs are subdivided according to the structure of their protein backbone into classical, lysine-rich, AG peptide, fasciclin-like (FLAs), non-classical, and chimeric AGPs [2,7,8]. The fasciclin-like arabinogalactan proteins (FLAs) are characterized by having fasciclin (FAS) domains which were first detected in fruit flies (*Drosophila melanogaster*) and later in other organisms including algae, lichens, plants, and animals. FLAs are found in numerous species. Twenty-one FLAs have been identified in *Arabidopsis thaliana* [9], 30 in *Musa spp*. [10], 33 in *Brassica rapa* L. [11], 23 in *Cannabis sativa* L. [12], 19 in *Corchorus olitorius* [13], 19 in *Gossypium hirsutum* [14], 18 in *Eucalyptus grandis* [15], 38 in *Nicotiana benthamiana* [16], 35 in *Populus trichocarpa* [17], 27 in *Oryza sativa* [3], 34 in *Triticum aestivum* [18], and 17 in *Cuscuta campestris* [19].

Studies on plant FLAs have focused on tissue-specific functions, pollination, and embryogenesis, as well as general responses to biotic and abiotic stresses [6,15,17,20]. In addition, numerous studies have demonstrated the importance of FLAs in aspects related to cell wall biosynthesis. For example, in Arabidopsis, *AtFLA4* induced aberrant cell expansion, adhesion, cell wall synthesis, and seed coat pectin mucilage [21]. *AtFLA11* and *AtFLA12* played a role in stem tensile strength, biomechanics, and elastic modulus, thus affecting the composition and structure of the cell walls [20]. Studies of *AtFLA16* mutants showed that deletion of *AtFLA16* resulted in reduced stem length and altered biomechanical properties [22]. Studies on poplar showed that the *PtFLA6* gene was associated with the poplar’s xylem fiber cells, cell wall composition, and stem biomechanics [23,24]. Woody plants have more developed lignified stems than herbaceous plants, and their lignified stems are mainly composed of secondary wall-thickened cells. Therefore, the prediction and study of FLA gene functions in woody plants could provide essential clues to improve the properties of wood.

*Populus* and *Salix* are sister genera belonging to the Salicaceae family. They are useful species for maintaining the ecological environment and for solving the shortage of wood [25,26]. Although the FLA gene family has been identified and analyzed in poplar [17], it has not been analyzed in willow. Here, we performed a genome-wide comparative analysis of the FLAs in *Populus* and *Salix*. A multi-species phylogenetic tree of the FLA gene family to explore differences in gene expansion in herbaceous and woody plants was constructed. The expression patterns of FLA genes in different tissues of both poplar and willow were combined and those that were strongly and specifically expressed during stem lignification and seed hair development were identified, thus providing reliable candidate genes for subsequent verification of the gene functions.

## 2. Results and Discussion

### 2.1. Genome-Wide Identification and Sequence Analysis of Populus and Salix FLA genes

Based on the sequence structural characteristics of the FLA gene family, 40 and 46 FLA gene family members were identified in the genomes of *P. deltoides* and *S. suchowensis*, respectively. They were named *PdeFLA1*-*PdeFLA40* and *SsuFLA1*-*SsuFLA46*, respectively, according to their positions on the chromosomes. All the genes contained the conserved FLA structural domain (Supplementary Tables S1 and S2). In *P. deltoides*, 29 proteins were predicted by the BIG-PI online program to have C-terminal glycophosphatidylinositol (GPI) anchors, and 34 N-terminal signal peptides were predicted by the SignalP online program (Supplementary Table S3). The proteins contained between 176 and 466 amino acids, with isoelectric points in the range of 4.50–10.77 and molecular weights of between 18,211.68 and 51,122.1 Da. In *S. suchowensis*, 32 proteins were predicted to have C-terminal GPI anchors by the BIG-PI prediction software, and 41 to were predicted to have N-terminal signal peptides by the SignalP online program (Supplementary Table S4). The amino acid lengths ranged from 206 to 468, the isoelectric points ranged from 4.33 to 10.81, and the molecular weights were between 21,708.68 and 51,646.93 Da. The gene names, gene IDs, chromosomal location, amino acid lengths, isoelectric point (pIs), and molecular weights (Mw) are listed in Table 1. This study comprehensively analyzed the FLA gene family in *P. deltoides* and *S. suchowensis*, identifying 40 and 46 genes, respectively. These gene numbers were 1.9-fold and 2.2-fold higher than the number of FLA genes in Arabidopsis [9]. Previous research has shown that early flowering species have a faster replacement rate than species with long generations [27,28]. This suggests that these two species have more genes than Arabidopsis, either because the poplar and willow genomes were larger or the replacement rate was much lower.

### 2.2. Chromosomal Distribution and Identification of Gene Duplication in Populus and Salix FLA Genes

**Figure 1:**
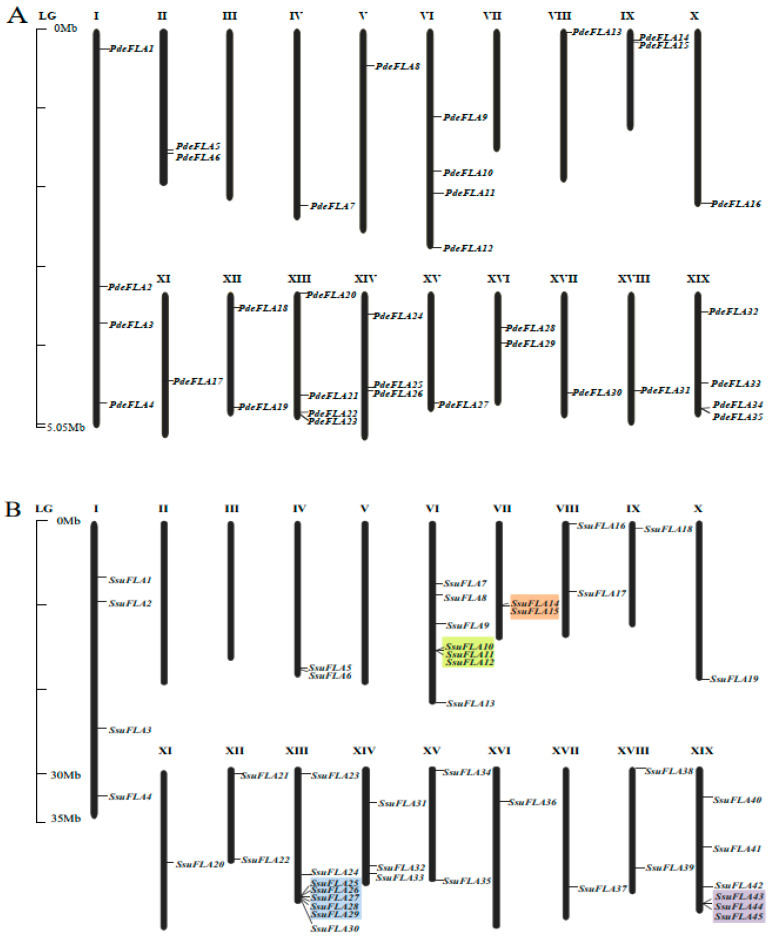
Location of FLA genes on chromosomes. Distribution of FLA gene family members on chromosomes in *P. deltoides* (**A**) and *S. suchowensis* (**B**). Genes are named according to their order on the chromosomes. Identified tandem repeat genes are highlighted using a colored background.

To determine the distribution of FLA gene family members on the chromosomes of the *P. deltoides* and *S. suchowensis* genomes, and to evaluate the gene duplication relationships among the genes, we mapped the 40 poplar and 46 willow FLA genes onto the 19 chromosomes of their respective genomes using nucleotide sequence alignment. Four poplar genes and two willow genes failed to map to chromosomes (Figure 1), but were mapped to unassembled scaffold fragments, probably due to incomplete genome assembly in these plant tissues.

**Figure 2:**
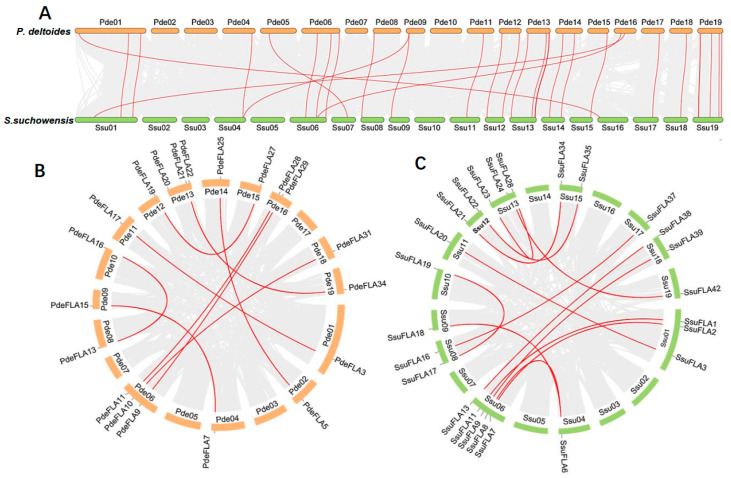
Synteny analysis of FLA genes. Segmental duplication of FLA gene pairs in *P. deltoides* (**A**) and *S. suchowensis* (**B**), with red lines indicating the correspondence of duplicated genes. (**C**) Gene duplication and synteny relationship of FLA genes between *P. deltoides* and *S. suchowensis*. The red lines indicate the syntenic FLA gene pairs, and the light gray indicates collinear blocks.

We found that the distribution of FLA genes on the chromosomes in the two studied genomes was uneven. The maximum number of FLA gene members distributed on the poplar chromosomes I, VI, XIII, and XIX was four, and no FLA genes were distributed on chromosomes III and VII (Figure 1A). There were relatively more FLA genes distributed on willow chromosomes VI, XIII, and XIX, with 7, 8, and 6 members (Figure 1B), respectively. No FLA genes were distributed on chromosomes II, III, and V. It was also found that the same number of FLA genes were distributed in the same positions on chromosomes I, X, XI, XII, XIV, and XVII of the two genomes, and it was speculated that they were orthologous genes. We analyzed the orthologous relationship between *P. deltoides* and *S. suchowensis*. Thirty-six pairs of orthologous genes were identified (Supplementary Table S5). The synteny analysis showed that 28 pairs of FLA genes were distributed in the same chromosomal position of *P. deltoides* and *S. suchowensis*, five pairs of genes were distributed in different positions, and three pairs of genes could not be determined linearly at the time because they were not yet mounted to the chromosome (Figure 2A). Our results demonstrated that the distribution of FLA genes is similar overall on both genomes, although there were large distribution differences on certain chromosomes. For example, compared with the poplar genome, there were no genes on chromosome II in the willow genome (Figure 1B), which might be because the evolution of willow species was faster than that of poplar species, and some genes that were not adapted to the environment were gradually eliminated.

Segmental and tandem duplication are the main drivers of gene family expansion. We found four pairs of tandem duplicated genes (*SsuFLA10*, *SsuFLA11* and *SsuFLA12*; *SsuFLA14* and *SsuFLA15*; *SsuFLA25*, *SsuFLA26*, *SsuFLA27*, *SsuFLA28* and SsuFLA29; and *SsuFLA43*, *SsuFLA44*, and *SsuFLA45*) in *S. suchowensis*, distributed on chromosomes VI, VII, XIII, and XIX (Figure 1B). No tandem duplicated genes were found in *P. deltoides*. Twelve segmental duplication events were identified in *P. deltoides*, involving 23 genes (Figure 2B and Supplemental Table S6). Thirteen segmental duplication events were identified in *S. suchowensis*, involving 25 genes (Figure 2C and Supplemental Table S6). All gene pairs, apart from *SsuFLA43/45* in *S. suchowensis*, which showed complete sequence identity, had Ka/Ks values less than 1 (Supplemental Table S6), indicating that the FLA gene family underwent purifying selection. A comparison of the gene duplication results between poplar and willow showed that the number of segmental duplication events was similar, but the location of segmental duplication differed greatly, with only six segmental duplications having the same chromosomal location. In addition, tandem duplication of FLA genes was more frequent in willow than poplar. The above results suggest that FLA genes may have undergone six segmental duplications within the common ancestral species of poplar and willow. After divergence of the two species, the average rate of gene replacement was significantly higher in the willow genome than in the poplar genome [27,28], and the rate of evolution was also higher [26,29]. This rapid expansion allowed the preservation of these newly generated species. This may also be why willow trees had more FLA genes than poplars.

### 2.3. Phylogeny, Conserved Gene Structures, and Protein Motif Analysis of Populus and Salix FLA Genes

**Figure 3:**
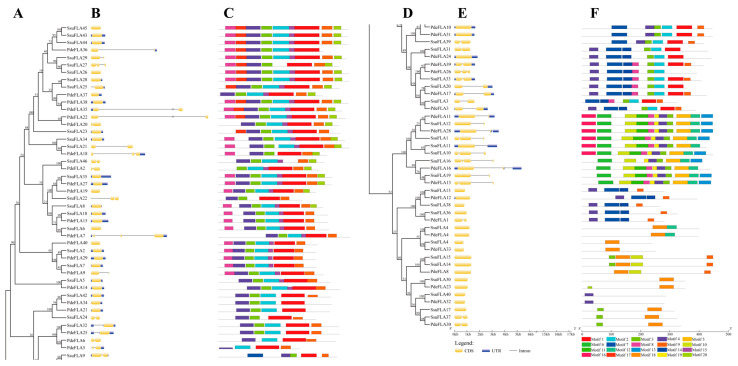
Phylogenetic relationships, gene structures, and motif compositions of FLA genes in *P. deltoides* and *S. suchowensis*. (**A**) Phylogenetics of FLA genes. (**B**) Genetic structure of exons and introns in each FLA gene. The yellow boxes represent exons and the single black lines represent introns. (**C**) The conserved motifs contained in each FLA protein. (**D**) Continuation of (**A**). (**E**) Continuation of (**B**). (**F**) Continuation of (**C**). Each motif is indicated using colored boxes and single black lines indicate non-conserved sequences. The bottom scale allows estimation of the size of each motif.

Both poplar and willow evolved from the same ancestral species, and during their respective evolution after species divergence, both retained orthologous genes inherited from their ancestors. At the same time, replication occurred in both genomes, generating new paralogous genes. To compare the sequences and structures of these homologous genes, we constructed a phylogenetic tree based on the full-length sequences of the FLA genes. We combined gene structure and conserved sequence prediction to present a comprehensive picture of the homology of FLA genes between *P. deltoides* and *S. suchowensis*. The analysis showed that there were 22 pairs of *P. deltoides* and *S. suchowensis* FLA genes at the extreme ends of the evolutionary tree branches, including *PdeFLA2* and *SsuFLA46*, *PdeFLA35* and *SsuFLA27*, and *PdeFLA18* and *SsuFLA15* (Figure 3A). In addition, multiple willow FLA genes were found to cluster with one poplar FLA gene on one evolutionary branch (Figure 3A). For example, *SsuFLA43*, *SsuFLA44*, and *SsuFLA45* clustered with *PdeFLA36* on the same branch, and this, combined with their chromosomal distributions, confirmed that *SsuFLA43*, *SsuFLA44*, and *SsuFLA45* were duplicates of genes generated by tandem repeats during willow evolution.

Normally, the FLA binding domain shows that introns were lost during plant evolution. Nevertheless, in our analysis, we found that 34.88% (30/86) of *P. deltoides* and *S. suchowensis* FLA genes contained one intron, 11.63% (10/86) had two introns, and 2.33% (2/86) had more than two introns, with only 51.16% (44/86) having no introns (Figure 3B). Overall, the intron structure varied widely among the FLA gene family members. However, combined with the phylogenetic tree analysis, our results indicated that genes on the same evolutionary branches of the *P. deltoides* and *S. suchowensis* FLA gene families had similar intron patterns. Furthermore, while some of the closest genes showed similar structures, a small subset showed different intron-exon arrangements. For example, *SsuFLA6* had no introns, while its nearby homologous gene, *PdeFLA7*, contained three, even though their evolutionary relationships reached 99% bootstrap values, respectively.

We further predicted the conserved motifs in the *P. deltoides* and *S. suchowensis* FLA proteins using the MEME online program to determine the specific regions of the FLA proteins. A comparison of the 20 conserved motifs found in *P. deltoides* and *S. suchowensis* (Figure 3C) revealed that the major motifs of the two species were similar, and the composition of the motifs was not significantly different. The lengths of the 20 conserved motifs ranged from 11 to 50 amino acids (Supplementary Table S7), and the number of motifs in each FLA protein ranged from 1 to 11. Most FLA proteins had motifs 1, 2, 3, 4, 8, and 9 (Figure 3C). Motifs 1, 2, 3, and 4 were included within the fasciclin domain, and motifs 8 and 9 were observed in the AGP-like glycosylation regions. However, while motifs 1, 2, 3, and 4 followed the fasciclin domain in the sequence and were particularly conserved in most clades, some motifs were selectively distributed between specific clades of the phylogenetic tree. For example, *PdeFLA32* and *SsuFLA40* contained only motif 4, and *SsuFLA41*, *SsuFLA30*, and *PdeFLA33* contained only motif 18. These results suggested that these conserved motifs played key roles in specific or similar functions.

### 2.4. Phylogenetic Analysis and Functional Prediction of the FLA Gene Family

**Figure 4:**
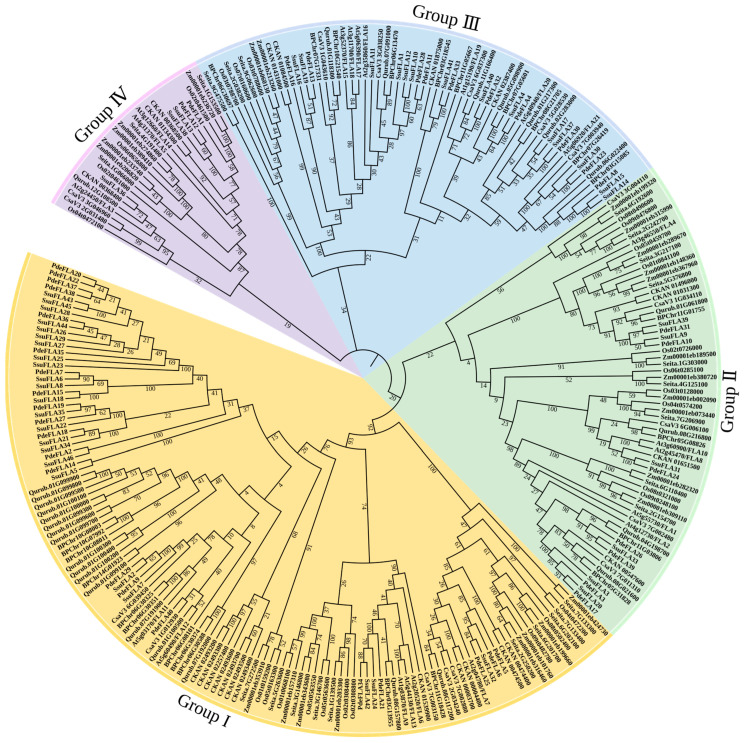
The phylogenetic tree of FLA proteins from *P. deltoides*, *S. suchowensis*, *Corylus avellana* L., *Quercus rubra* L., *Cinnamomum kanehirae*, *Setaria italica*, *Zea mays* L., *Cucumis sativus* L., *Oryza sativa* L., and *Arabidopsis thaliana*. A total of 277 FLA proteins were analyzed using ClustalX 2.1, and the Neighbor-Joining tree was constructed using MEGA 11 software. The bootstrap value was 1000 replicates. The FLA proteins were clustered into four distinct groups (I–IV), represented by different colors.

We have investigated the phylogenetic relationships of the FLA proteins in different plants and constructed a phylogenetic tree including five woody plants (*P. deltoides*, *S. suchowensis*, *Betula platyphylla*, *Quercus rubra* L., and *Cinnamomum kanehirae*) and five herbaceous plants (*Setaria italica*, *Zea mays* L., *Cucumis sativus* L., *Oryza sativa* L., and *Arabidopsis thaliana*). In all, 277 FLA gene members were classified using the neighbor-joining (NJ) method. The phylogenetic tree showed that the FLA gene family was divided into four evolutionary branches, which were named Group I, II, III, and IV, respectively (Figure 4).

The statistics of the gene members in each group showed that Group I was the largest group, containing 123 genes, while Group IV was the smallest, with 26 genes. The number of genes in each group was counted for each species used in the construction of the evolutionary tree, and it was found that the woody plants had more Group I genes than herbaceous plants. Group I genes made up an average of 50.49% of the FLA genes in woody plants, while the average proportion in herbaceous plants was 33.16% (Supplementary Table S8). Additionally, Group I FLA made up an average of 0.04584% of all genes in the genomes of woody plants, but only 0.02118% in herbaceous plants (Supplementary Table S8). The differences in these proportions between woody and herbaceous plants were found to be highly significant (Supplementary Figure S1). This suggests that among the plants selected for evolutionary analysis, FLA genes in the Group I branch of woody plants underwent a major expansion. It also implies that FLA genes play important roles in secondary growth in woody plants.

Combined with the functional conservation of homologous genes, genes with similar or identical functions were classified into the same groups, providing a reliable reference basis for predicting the functions of related genes in the gene family. To predict the biological functions of the poplar and willow FLA genes, we referred to the functions of FLA genes that have been verified in Arabidopsis. The gene members in Group I belonged to the same subgroup as the *AtFLA11* and *AtFLA12* genes that are associated with xylem development. Related studies have found a correlation between the abundance of *AtFLA11* and *AtFLA12* transcripts containing a single FAS domain and the onset of secondary cell wall cellulose synthase expression in *Arabidopsis* stems [30,31]. In addition, the phenotypes of *AtFLA11* mutants showed the presence of a mild collapsed vessel phenotype and reduced stem cellulose content [30,31,32]. These analyses indicated that the FLA members in Group I were associated with secondary wall and cellulose synthesis in the stem. Group II proteins fall into the same subgroup as *AtFLA1*. Several studies have described a T-DNA insertion mutant in the *AtFLA1* gene under standard growth conditions, showing that although the FLA1-1 mutants had no distinct phenotype, the gene may play a role in the ability of the callus to induce regeneration, shown by in vitro shoot induction assays [33]. Therefore, we speculate that the Group II proteins might be involved in the development of buds. *AtFLA16* belongs to Group III, and studies on *AtFLA16* mutants showed that the deletion of the gene resulted in reduced stem lengths and altered biomechanical properties [22]. We thus speculate that Group III members are responsible for maintaining and regulating stem strength and support. Group IV includes *AtFLA3*, and overexpression of the gene led to defective elongation of stamen filaments and reduced female fertility resulting in siliques with low seed settings, suggesting that *AtFLA3* was involved in microspore development and might affect the pollen lining by participating in cellulose deposition [34]. Therefore, we speculate that the members of Group IV might have functions involved in the development of microspores.

A combination of the phylogenetic analysis and functional predictions of FLA genes strongly suggested that genes in Group I were likely to be associated with cell wall synthesis and secondary growth in plants. In particular, two sets of tandem repeat genes from willow (*SsuFLA10*, *SsuFLA11*, and *SsuFLA12* and *SsuFLA43*, *SsuFLA44*, and *SsuFLA45*) were included in Group I (Figure 4). In addition, it has been shown that a subset of FLAs containing a single FAS domain contributed to plant stem strength by affecting cellulose deposition. It has also been suggested that this influences the stem elastic modulus by affecting the integrity of the cell wall matrix [20]. We further found that FLA genes in Group I contained only one FAS domain and had undergone duplication. Thus, these analyses supported our hypothesis that Group I genes were involved in plant cellulose and cell wall formation, were significantly amplified in woody plants, and were involved in wood formation. These results suggested that these genes were good candidates for further functional verification and phylogenetic analysis.

### 2.5. Identification of FLA Candidate Genes Related to Stem Development in Populus and Salix

Wood production relies on the activity of vascular bundle-forming layers. A primary vascular system consists of a discrete set of vascular bundles that contain bundle-forming layers, primary phloem, and primary xylem. The primary vascular bundle originates from proto-formation layer cells on the periphery of the rib region of the shoot apical meristem (SAM). In perennial woody plants, secondary growth arises from the meristem activity of the vascular formation layer, and the bundle-like formation layer located at the center of the primary vascular bundle extends to the interbundled region, tangentially generating an interbundled formation layer to form a vascular formation layer ring [35,36]. The meristem activity of the vascular forming layer then leads to the continuous production of cylindrical secondary vascular tissue (wood). Therefore, we selected tissues with different degrees of lignification in *P. deltoides* and *S. suchowensis* stems and analyzed the expression patterns of Group I members in the FLA gene family. Since some genes had high sequence similarity, it was impossible to design specific primers to distinguish them, so universal primers were designed for these genes to analyze their expression patterns. For example, *PdeFLA19* and *PdeFLA27* were 91.42% similar; *PdeFLA35* and *PdeFLA38* were 96.09% similar; *SsuFLA43*, *SsuFLA44* and *SsuFLA45* were 97.99% alike; and *SsuFLA25*, *SsuFLA26* and *SsuFLA28* were 93.38% identical.

**Figure 5:**
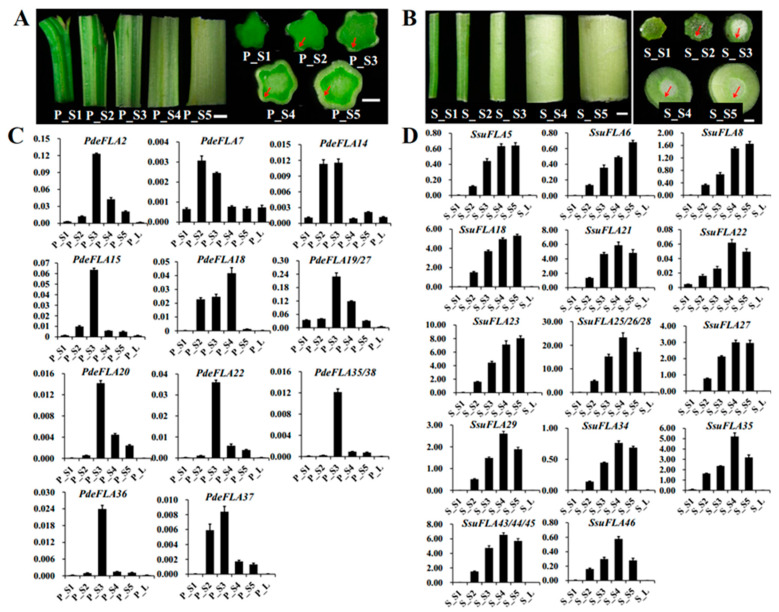
Expression analysis of Group I members of the FLA gene family in *P. deltoides* and *S. suchowensis* at different stages of stem lignification development. (**A**,**B**) Observations of *P. deltoides* and *S. suchowensis* at stem lignification development sites. The red arrow points to the xylem. Scale bar = 0.5 mm. (**C**,**D**) Expression pattern analysis of Group I members in *P. deltoides* and *S. suchowensis*. The *X*-axis represents different tissues and the *Y*-axis represents relative expression. Values shown are means ± SD of three biological replicates.

In *P. deltoides*, five parts of the stem, internodes 1-2, 3-4, 5-7, 8-9, and 10-11, were selected from the stem tip downwards and named P_S1, P_S2, P_S3, P_S4, and P_S5, respectively (Figure 5A). Expression patterns were analyzed for members of some FLA gene families in Group I. RT-qPCR analysis showed (Figure 5C) that *PdeFLA2/15/19/20/22/27/35/36/38* were expressed significantly in the elongated region of the stem (P_S3) but were significantly reduced in the tip of the stem (P_S1) and the secondary growth region of the stem (P_S4). On the other hand, *PdeFLA7/14/37* was expressed mainly in young stems (P_S2) and elongated regions of stems (P_S3). In particular, *PdeFLA19/27* had the highest relative expression. These results suggested that *PdeFLA19/27* was associated with secondary thickening growth and could thus serve as a major candidate gene for later studies on poplar wood growth and development.

We selected tissues from *S. suchowensis*, as we did in poplar. They were named S_S1 to S_S5 and were analyzed by RT-qPCR to determine the expression patterns of the Group I FLA family members. The results of the stem cross-sections showed that the willow had a faster lignification rate than the poplar in the same nodal tissues, especially in S4 and S5, where the secondary vascular tissue formed a thicker structure (Figure 5B). The RT-qPCR analysis showed that a total of 17 genes (Figure 5D), all members of the Group I branch in willow, were positively correlated with stem node lignification and had a very low expression in shoot leaves and stem tips (S_S1). The expression of *SsuFLA5/6/18/23/27* increased gradually from S_S1 to S_S5, while the expression levels of Ssu*FLA21/22/25/26/28/29/34/35/43/44/45/46* were highest in S_S4 tissue, but tended to decrease in S_S5 tissue (Figure 5D). Therefore, it was inferred that rapid lignification of willow stems occurred at internodes 8-9 (S_S4). The Group I FLA genes showed greater functional redundancy in the regulation of stem lignification function. Of these, the three tandem repeats of *SsuFLA25/26/28* were expressed at high levels and played a dominant role in willow stem lignification. These results suggested that these could be important candidate genes for further investigation of wood development and formation in willow.

### 2.6. Identification of FLA Candidate Genes Related to Seed Hair Development in Populus and Salix

**Figure 6:**
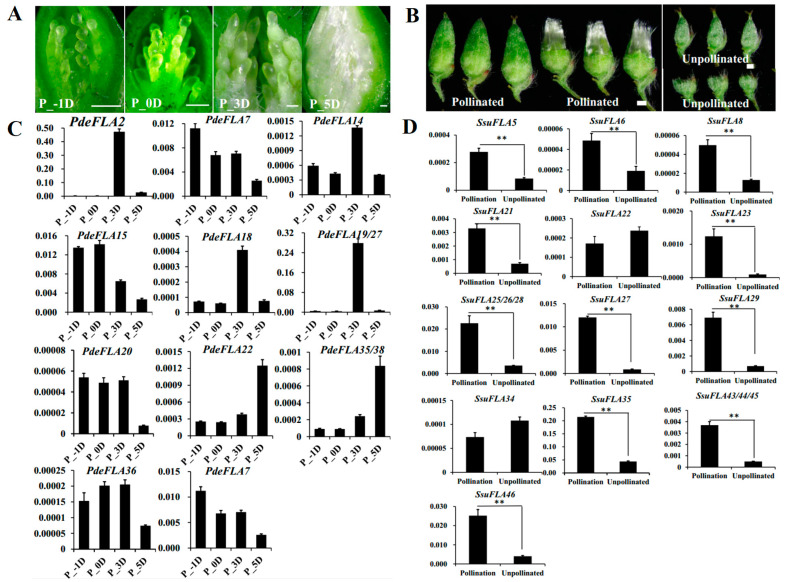
Analysis of the expression of *P. deltoides* and *S. suchowensis* FLA Group I genes involved in seed hair development. (**A**) Observation of different stages of seed hair development in *P. deltoides*. Scale bar = 0.5 mm. (**B**) Observation of seed hair phenotypes of *S. suchowensis* florets in pollinated versus unpollinated treatments. Scale bar = 0.5 mm. (**C**,**D**) Expression patterns of Group I FLA genes in *P. deltoides* and *S. suchowensis* (*p*-values are provided in Supplementary Table S9). The *X*-axis represents different tissues and the *Y*-axis represents relative expression. Values shown are means ± SD of three biological replicates (** *p*  <  0.01, two-sided Student’s *t*-test).

Seed hairs are composed of flocculent fibers produced by poplar and willow trees to assist in seed dispersal after reaching sexual maturity. They are formed by the projection of placentation epidermal cells and their cell walls are largely composed of cellulose, hemicellulose, and pectin. As in the investigation of the regulatory roles of FLA genes in plant cell walls, we collected tissues from different periods of poplar seed hair development to analyze the expression of the Group I genes. The results showed that poplar seed hairs produced obvious flocculent fibers that elongated rapidly 3 days after pollination (DAP). Furthermore, the length of seed hairs was sufficient to completely wrap the ovules by 5 DAP, indicating that the 3–5 DAP period was associated with the rapid elongation of seed hairs (Figure 6A).

Furthermore, the RT-qPCR analysis showed that *PdeFLA2/18/19/27* genes were highly expressed specifically at 3 DAP, which was consistent with the rapid elongation development of seed hairs. The highest relative expression was seen in the *PdeFLA19/27* genes (Figure 6C). We therefore speculate that these genes have important regulatory roles in the development of cell walls during the rapid elongation of poplar seed hairs.

In *S. suchowensis*, we collected pollinated and unpollinated florets in the same catkin to analyze the expression of Group I FLA genes. The results showed that pollinated florets developed numerous seed hairs on 7 DAP, while unpollinated florets did not produce seed hairs (Figure 6B). Furthermore, RT-qPCR analysis showed that 15 of the 17 gene Group I genes were expressed at significantly higher levels in pollinated florets than in unpollinated tissue, except for *SsuFLA22/34* (Figure 6D). Of these, the *SsuFLA35* gene showed the highest relative expression in pollinated florets, and it is speculated that it may be involved in regulating the development of willow seed hair tissue.

## 3. Materials and Methods

### 3.1. Genome-Wide Identification and Sequence Analysis of Populus and Salix FLA Genes

The genome sequences of *P. deltoides* (PRJNA598948) and *S. suchowensis* (ASM1755242v1) were downloaded from the NCBI website (https://www.ncbi.nlm.nih.gov/ accessed on 8 March 2021). The fasciclin domain genome sequence (PF02469) was downloaded from the Pfam database (http://pfam.xfam.org/ accessed on 8 March 2021). HMM3.0 software was used to screen proteins containing this domain in the genome sequences of *P. deltoides* and *S. suchowensis* [37]. In addition, a local database was constructed from the whole genome sequences of *P. deltoides* and *S. suchowensis*, and a BLASTP search was performed using an FLA protein sequence of *A. thaliana*. The candidate protein sequences identified by the above two methods were combined to remove duplicates, incomplete sequencing, and protein sequences without complete coding frames. Candidate proteins were analyzed using the Pfam (http://pfam.xfam.org/ accessed on 8 March 2021, PF02469) [38] and SMART (http://smart.embl.de/ accessed on 8 March 2021) [39] databases, and protein sequences with incomplete domains were removed.

Prediction of the N-terminal signal peptides was performed on the SignalP 5.0 website (https://services.healthtech.dtu.dk/service.php?SignalP-5.0 accessed on 8 March 2021) [40]. Prediction of the attachment sites of the C-terminal GPI anchor was performed in the BIG-PI database (http://mendel.imp.ac.at/sat/gpi/gpi_server.html accessed on 8 March 2021) [41]. The fasciclin domain, N-terminal signal peptide, and C-terminal GPI anchor attachment site were removed for further screening for AGP-like glycosylation sites. Finally, the sequences containing the fasciclin domain and AGP-like glycosylation regions were considered the *P. deltoides* and *S. suchowensis* FLA gene family sequences. ExPASy (https://web.expasy.org/compute_pi accessed on 8 March 2021) was used for the prediction of the pI (isoelectric point) and molecular weight (Mw) [42].

The nucleotide and protein sequences of the identified FLA gene family members were aligned with the whole genome sequences of *P. deltoides* and *S. suchowensis* using BLAST, and the corresponding genome sequences, chromosomal positions, and exon/intron distribution patterns of each member were obtained. In addition, the genome sequences of six other species were downloaded from different websites, including *Corylus avellana* (https://hardwoodgenomics.org/bio_data/4302786?tripal_pane=group_downloads accessed on 14 May 2021), *Quercus rubra* L. (https://phytozome-next.jgi.doe.gov/info/Qrubra_v2.1 accessed on 14 May 2021), *Cinnamomum kanehirae* (https://phytozome-next.jgi.doe.gov/info/Ckanehirae_v3 accessed on 14 May 2021), *Setaria italica* (https://phytozome-next.jgi.doe.gov/info/Sitalica_v2.2. accessed on 14 May 2021), *Zea mays* L. (http://plants.ensembl.org/Zea_mays/Info/Index accessed on 14 May 2021), and *Cucumis sativus* L. (http://cucurbitgenomics.org/ftp/genome/cucumber/Chinese_long/v3/ accessed on 14 May 2021), and their respective FLA gene families were identified. Members of the FLA gene family of *A. thaliana* (21 sequences) [9] and *Oryza sativa* L. (27 sequences) [3] were also downloaded (Supplementary Sequence S1).

### 3.2. Chromosomal Distribution of FLA Genes

Using the annotation files of the *P. deltoides* and *S. suchowensis* genomes, the chromosome location information of the FLA gene family was extracted, and the length of each chromosome was obtained. MapChart software was used to map the chromosomal locations and relative distances in the FLA genes. Tandem duplications of FLA genes were confirmed based on two criteria: (1) genes distributed in the 100 kb range on chromosomes and (2) similarity of >70% of the sequence alignment of the two genes [43,44]. Synteny analysis between species and gene duplication within species were performed using MCScanX software. Visual mapping was completed using Tbtools and KaKs_Calculator 2.0 was used to calculate synonymous (Ks) and non-synonymous (Ka) substitution of FLA gene pairs.

### 3.3. Phylogenetic Analysis and Functional Prediction of FLA Genes

Multiple sequence comparisons of FLA full-length protein sequences of *P. deltoides* and *S. suchowensis* were performed using ClustalX 2.1 with the default parameters [45]. Neighbor-joining (NJ) phylogenetic trees were constructed using MAGE 11 with parameters set to Poisson correction, pairwise deletion, and bootstrap analysis with 1000 replicates [46]. All FLA protein sequences from *P. deltoides*, *S. suchowensis*, *C. avellana*, *Q. rubra* L., *C. kanehirae*, *S. italica*, *Z. mays* L., *C. sativus* L., *O. sativa* L., and *A. thaliana* were clustered by MEGA 11 software for multiple sequence alignment and construction of the phylogenetic tree. The phylogenetic tree was visualized using the online program iTOL (https://itol.embl.de/login.cgi accessed on 11 August 2021), and a folded phylogenetic tree consisting of 10 species was constructed [47].

### 3.4. Analysis of Gene Structure and Protein Motif Identification

The intron and exon positions of the FLA genes were extracted, and the number and length of introns and exons were counted in generic feature format (GFF) files released for the whole genomes of *P. deltoides* and *S. suchowensis*. The exon and intron structures were displayed through the online program Gene Structure Display Server (http://gsds.gao-lab.org/index.php accessed on 8 March 2021) [48]. The MEME online program (https://meme-suite.org/meme/tools/meme accessed on 8 March 2021) was used to identify conserved motifs in the FLA proteins. The applied inclusion criteria were: (1) each FLA protein sequence appeared with at least 1 and a maximum of 20 motifs; (2) each motif had a minimum of 2 and a maximum of 250 amino acids in the sequence; and (3) other default parameters were used. The IDs of FLA members were kept the same as previously named.

### 3.5. Preparation of Plant Materials

Stems with different degrees of lignification were collected. Six-month-old *P. deltoides* and *S. suchowensis* were selected at the Plant Growth Laboratory of Nanjing Forestry University (China, Nanjing). Five parts of the stem, internodes 1–2, 3–4, 5–7, 8–9, and 10–11, were selected from the stem tip downwards and named S1, S2, S3, S4, and S5. Tissues at different stages of poplar seed hair development were also collected; all were from female *P. deltoides* flowering branches. Hydroponics was used to promote flowering. Pollination was performed with the stigma fully expanded, termed 0 days after pollination (0 DAP). Inflorescence ovaries were collected at −1 DAP, 0 DAP, 3 DAP, and 5 DAP in sequence. For the collection of willow seed hair tissue, male and female flowering branches of *S. suchowensis* were selected to promote flowering using hydroponics. After opening of the female inflorescences, some of the florets were pollinated. Seven days after pollination, pollinated and unpollinated florets in the same inflorescence were collected separately. Three replicates of all samples were collected and were immediately frozen in liquid nitrogen after collection and stored at −80 ℃ for RNA extraction.

### 3.6. Primer Design, RNA Extraction, and qRT-PCR

The primers used for analyzing the expression of FLA genes in *P. deltoides* and *S. suchowensis* were designed using Primer 5.0. The specificity of the primers was verified by agarose gel electrophoresis and the primer sequences are shown in Supplementary Table S10. *PtUBQ* (Potri.015G013600.1) [49] and *ACT7* (SapurV1A.0231s0320.1) [50] were used as internal reference genes for *P. deltoides* and *S. suchowensis*, respectively. Total RNA was extracted from the *P. deltoides* and *S. suchowensis* tissues using the RNA Easy Fast Plant Tissue Kit (TIANGEN, Nanjing, China). One microgram of RNA from each sample was reverse transcribed to cDNA using the One-Step gDNA Removal and cDNA Synthesis SuperMix (TIANGEN, Nanjing, China). Quantitative RT-PCR was performed on a 7500 Fast Real-Time PCR System (Applied Biosystems, USA). The amplification reaction system was as follows: 1 µL each of forward and reverse primers (10 umol/L), 2 µL cDNA (5-fold dilution), 10 µL PowerUpTM SYBRTM Green Master Mix (Applied Biosystems), and RNase-free water was added to make the solution up to 20 µL. The amplification procedure was as follows: 95 °C for 15 s, 60 °C for 30 s, and 72 °C for 30 s, with 40 cycles. Finally, the relative expression of each FLA gene was calculated using the CT method [51].

## 4. Conclusions

In this study, we performed a systematic analysis of the FLA gene family in *P. deltoides* and *S. suchowensis*, including the identification of the FLA genes and analysis of their structure and chromosomal distribution, as well as the identification of conserved domains and the evolution of the gene family in multiple species. The analysis revealed that FLA genes on the Group I branch of the phylogenetic tree were involved in the regulation of secondary growth processes, such as lignification in poplar and willow, and further showed that these genes had undergone significant expansion in woody plants, suggesting that this group of genes may have been important in the evolution of woody plants from herbaceous plants. Finally, analysis of the expression of the Group I genes in different tissues confirmed that the *PdeFLA19/27* gene was highly expressed in poplar, specifically during stem lignification and seed hair development, suggesting its important regulatory role in stem or seed hair development. Furthermore, it was verified that a set of duplicated genes, *SsuFLA25/26/28,* in willow were important candidates for stem lignification development, and that *SsuFLA35* may be involved in the regulation of germplasm hair development. In summary, this study priovides a multi-faceted reference for subsequent studies on the functions of FLA genes in poplar and willow.

## Figures and Tables

**Table 1:** Putative FLA genes identified in the genomes of *P. deltoides* and *S. suchowensis*.

Gene Symbol	Gene ID	Chr	Start	End	Amino Acid	pI	Mw
*PdeFLA1*	EVM0033614	chr1	2863,934	2,864,908	281	7.79	28,804.55
*PdeFLA2*	EVM0013530	chr1	31,953,859	31,954,589	213	5.58	22,696.96
*PdeFLA3*	EVM0016507	chr1	37,804,827	37,807,480	406	5.49	43,144.42
*PdeFLA4*	EVM0019309	chr1	46,418,162	46,419,355	397	5.06	42,295.82
*PdeFLA5*	EVM0012430	chr2	14,552,755	14,553,795	176	6.38	18,211.68
*PdeFLA6*	EVM0003247	chr2	14,743,911	14,744,707	254	4.50	26,606.07
*PdeFLA7*	EVM0003107	chr4	20,721,639	20,727,689	323	8.59	34,386.37
*PdeFLA8*	EVM0000914	chr5	6,121,373	6,122,701	442	6.03	47,578.60
*PdeFLA9*	EVM0035476	chr6	10,905,263	10,906,724	227	4.75	24,112.12
*PdeFLA10*	EVM0035420	chr6	17,439,680	17,441,342	426	5.23	44,980.52
*PdeFLA11*	EVM0030864	chr6	20,717,497	20,720,696	466	6.07	50,934.06
*PdeFLA12*	EVM0005454	chr6	26,560,554	26,561,810	394	8.92	41,861.21
*PdeFLA13*	EVM0010041	chr8	598,740	601,878	440	5.98	48,356.80
*PdeFLA14*	EVM0021839	chr9	2,003,076	2,004,125	263	8.44	28,699.16
*PdeFLA15*	EVM0017387	chr9	2,005,847	2,007,233	269	8.40	28,404.59
*PdeFLA16*	EVM0034822	chr10	21,572,863	21,578,206	460	6.21	50,769.91
*PdeFLA17*	EVM0012659	chr11	11,492,405	11,495,560	408	5.69	43,677.05
*PdeFLA18*	EVM0036111	chr12	1,843,829	1,848,143	229	7.77	23,800.59
*PdeFLA19*	EVM0016248	chr12	14,176,837	14,177,559	240	5.33	25,352.79
*PdeFLA20*	EVM0035889	chr13	1,002,373	1,003,172	255	7.02	27,077.81
*PdeFLA21*	EVM0037601	chr13	13,975,549	13,976,510	241	6.55	25,114.65
*PdeFLA22*	EVM0020019	chr13	15,988,602	16,005,827	269	6.64	28,265.15
*PdeFLA23*	EVM0022991	chr13	16,027,081	16,028,142	353	9.32	39,230.62
*PdeFLA24*	EVM0034823	chr14	5,118,954	5,120,795	423	5.36	43,390.45
*PdeFLA25*	EVM0004019	chr14	12,708,764	12,710,721	262	5.56	27,645.30
*PdeFLA26*	EVM0001785	chr14	13,240,881	13,242,143	376	7.65	40,562.30
*PdeFLA27*	EVM0017855	chr15	13,612,965	13,614,288	240	6.72	25,379.90
*PdeFLA28*	EVM0008767	chr16	4,738,845	4,742,370	466	5.90	51,122.10
*PdeFLA29*	EVM0000252	chr16	7,252,981	7,254,142	239	5.73	25,172.56
*PdeFLA30*	EVM0030626	chr17	12,475,605	12,476,664	339	4.50	37,001.51
*PdeFLA31*	EVM0029903	chr18	11,808,901	11,810,496	427	5.29	44,971.60
*PdeFLA32*	EVM0013339	chr19	1,874,378	1,875,226	282	10.77	30,578.54
*PdeFLA33*	EVM0014374	chr19	9,899,008	9,899,766	252	7.76	27,151.55
*PdeFLA34*	EVM0017850	chr19	15,221,093	15,221,992	245	6.72	25,421.84
*PdeFLA35*	EVM0023928	chr19	17,054,292	17,069,477	261	6.50	27,443.27
*PdeFLA36*	EVM0017906	chr19	17,076,489	17,081,736	212	9.68	22,669.99
*PdeFLA37*	EVM0003708	Contig00307	137	987	211	6.18	22,266.27
*PdeFLA38*	EVM0009936	Contig00307	14,016	15,179	263	6.50	27,647.50
*PdeFLA39*	EVM0004735	Contig00342	28,127	29,763	397	5.51	42,607.59
*PdeFLA40*	EVM0030058	Contig02018	2408	3139	243	9.04	25,611.18
*SsuFLA1*	EVM0026394	chr1	6,636,804	6,639,178	454	6.29	49,805.69
*SsuFLA2*	EVM0024166	chr1	9,407,953	9,408,990	240	5.29	25,207.58
*SsuFLA3*	EVM0006591	chr1	25,816,885	25,817,328	340	5.19	35,939.01
*SsuFLA4*	EVM0006450	chr1	32,542,611	32,543,813	400	5.47	42,028.31
*SsuFLA5*	EVM0021770	chr4	15,902,015	15,902,939	242	8.89	26,127.29
*SsuFLA6*	EVM0006635	chr4	15,905,288	15,906,103	271	8.55	28,341.48
*SsuFLA7*	EVM0003490	chr6	8,865,503	8,866,458	241	6.55	25,146.69
*SsuFLA8*	EVM0000130	chr6	10,047,978	10,048,840	257	7.85	27,187.19
*SsuFLA9*	EVM0014792	chr6	12,547,684	12,549,068	388	5.16	40,792.62
*SsuFLA10*	EVM0022300	chr6	14,817,544	14,820,039	468	7.77	51,646.93
*SsuFLA11*	EVM0039496	chr6	14,859,433	14,862,830	444	6.06	48,952.77
*SsuFLA12*	EVM0030612	chr6	14,870,417	14,872,828	465	6.25	51,410.46
*SsuFLA13*	EVM0002771	chr6	20,137,156	20,138,004	282	8.98	29,097.38
*SsuFLA14*	EVM0038485	chr7	9,014,791	9,016,143	450	5.95	48,762.99
*SsuFLA15*	EVM0004828	chr7	9,076,360	9,077,712	450	5.95	48,775.05
*SsuFLA16*	EVM0004641	chr8	467,485	470,609	428	5.78	47,346.78
*SsuFLA17*	EVM0030085	chr8	7,324,477	7,325,427	316	4.68	34,441.38
*SsuFLA18*	EVM0025887	chr9	1,053,546	1,054,711	269	6.41	28,307.37
*SsuFLA19*	EVM0026596	chr10	16,349,980	16,352,801	461	5.61	50,880.71
*SsuFLA20*	EVM0032967	chr11	9,355,631	9,358,661	344	6.14	37,231.76
*SsuFLA21*	EVM0003643	chr12	927,908	931,248	275	6.41	28,692.16
*SsuFLA22*	EVM0039047	chr12	9,693,341	9,693,356	206	5.78	21,712.59
*SsuFLA23*	EVM0028769	chr13	792,822	793,782	257	7.74	27,166.11
*SsuFLA24*	EVM0029643	chr13	12,512,025	12,512,743	211	4.73	21,708.68
*SsuFLA25*	EVM0007673	chr13	14,193,258	14,194,071	245	6.51	25,665.23
*SsuFLA26*	EVM0039302	chr13	14,197,604	14,198,398	264	7.87	27,766.61
*SsuFLA27*	EVM0003847	chr13	14,200,804	14,202,010	245	8.67	25,785.40
*SsuFLA28*	EVM0021248	chr13	14,285,373	14,286,276	265	7.88	27,869.72
*SsuFLA29*	EVM0029154	chr13	14,289,871	14,290,923	259	7.06	27,479.26
*SsuFLA30*	EVM0019512	chr13	14,308,009	14,309,070	353	8.91	39,166.45
*SsuFLA31*	EVM0039706	chr14	4,440,309	4,441,573	411	5.37	42,242.28
*SsuFLA32*	EVM0024403	chr14	10,667,361	10,669,307	260	4.73	27,416.04
*SsuFLA33*	EVM0016971	chr14	11,035,380	11,037,015	391	7.09	41,605.74
*SsuFLA34*	EVM0008734	chr15	781,936	782,981	267	6.50	28,227.71
*SsuFLA35*	EVM0034733	chr15	11,756,924	11,758,526	240	6.57	25,349.91
*SsuFLA36*	EVM0008341	chr16	2,217,768	2,218,745	325	6.57	33,166.50
*SsuFLA37*	EVM0021655	chr17	10,904,748	10,905,794	325	4.33	35,075.06
*SsuFLA38*	EVM0000963	chr18	324,610	325,410	266	5.45	27,430.66
*SsuFLA39*	EVM0000740	chr18	9,619,792	9,620,553	397	4.96	42,023.18
*SsuFLA40*	EVM0022185	chr19	3,251,016	3,251,879	287	10.81	31,346.24
*SsuFLA41*	EVM0019293	chr19	9,639,274	9,639,996	240	8.41	25,832.92
*SsuFLA42*	EVM0007092	chr19	14,655,296	14,656,329	243	6.27	25,492.98
*SsuFLA43*	EVM0013439	chr19	16,018,464	16,019,599	263	6.97	27,756.63
*SsuFLA44*	EVM0030289	chr19	16,037,704	16,038,793	263	6.58	27,414.32
*SsuFLA45*	EVM0013597	chr19	16,041,720	16,042,511	263	6.97	27,756.63
*SsuFLA46*	EVM0006563	Contig04279	21,081	21,802	223	7.06	24,277.03

## Data Availability

Data is contained within the article or Supplementary Material.

## Supplementary Materials

The following supporting information can be downloaded at: https://www.mdpi.com/article/10.3390/ijms24021481/s1.
