# Supplementary material for: Genome-Wide Comparative Analysis of the Fasciclin-like Arabinogalactan Proteins (FLAs) in Salicacea and Identification of Secondary Tissue Development-Related Genes
Source: Int J Mol Sci. 2023 Jan 12;24(2):1481. doi: 10.3390/ijms24021481 (PMC9862198; doi:10.3390/ijms24021481)
Supplement: Supplementary file 1 [file ijms-24-01481-s001.zip › Supplemental Table S1.pdf]

Supplemental Table S1. Prediction of conserved structural domains of FLA proteins in *P. deltoids*.

Name	Precursor protein backbones
PdeFLA1	MSLKSSSLFALCFSSFLLFNTVRASNITQILSQYPDFSTFSSYLTQTQLAGEINSRQTITVLVVENGNMSPLSGKPNGEIKNVLSGHVILDYYDVAKLQKLQNKTAMLTTLFQSSGQAKGQQGFLNVTVLGANSVAFGSAVPGSSLSSNLVKSVSSQPYNISVLQVSNIIVPAGTGNTNSTTSPVPVGPPKTSPTPASSPNKPPSTSNPPPRPSPTTADGPAADSPAPSPPAMDGPIAATPAADGSLADAPSDLKSDASVINTGNNLALLALILLSAFVLA
PdeFLA2	MERLQHLLISLYLLILSINLTTITAQSPAPAPAPPGPTNLGNTNNGLTILAPSDSAFSALKTGTLRTLTDQEKVELMQFHIVPMFISSSQFDTVSSPLKTHAGSGARFQLNVTASGNSLNISTGLTNTTISDTVYTDTHLAIYQVDKVLLPLDIFTPKPPPPAPAPAPKLKEESESPDDAVSKKDISSAVSFVMHHDTVFFIVGTVVAISFSL
PdeFLA3	MQLTVLLSLLFLLSTATTITYGHNITSILGQHPSLSTFNHYLTLTHLAGEINRRTTITVCVVDNAAMSEILSKKPSISTIKNILSLHVLLDYFGTKKLHQIRDGTALAATMFQATGSAPGSTGFVNITDVKGGKVAFGPKDNGGNLDVFYVKSVEEIPYNISVIQISKLLPSDVAAAPTPEPSAMNITDIMSAHGCKVFADTLIANPDASKTYQDTIDGGLTVFCPLDDPFKAFLPKFKNLTASGKESLLQFFGVPVYQSLAMLKSNNGIMNTLATNGDKKFDFTVQNDGEDVTLKTRGTTAKIVGTLIDEQPLAIYSIDKVLLPKELFKAALTPAPAPAPEEAADAPKSSKHKKPSADDAPSDSPADSPDGDAADQTADDNASVRLDGGRLVAMVLSLCLGLLML
PdeFLA4	MAKYLFSFLLLLLTTTTATSTFNPICTFSPTPSPTSATTPAPTPITNTNTNIALSPTFAPSPIITIPTPAPSSAPTPINNTTTTTLTPTTTPSTAPTPIPTNKITLTPTFAPSLAPTPTIITPILGPSPAPTPISSSTFTTTPTFDPSPAPTPTNSSIFTATFSSLPPTPPSTFTPQQDDLKFVFQEQIYNIIDAILGTGDFKNWANALSMADSTTFPISATFFIPSDNSLSPITTSADPDIFPYHIVPQRLSFADLQQFKTFSRLPTLLFDKSILITNNSASHFTLDGSRLTHPDIYTNAAITVHGIDNLLDHSVYGTESGKNSSKPDAVGPPPTPASPPRPTPRTFVPSTADDEEFTVHQHGESDAACLCTEVWTVFLVLCVALASKFQRMILVH
PdeFLA5	MMGLCSICSKAASPPAPTPTPSSSPAPAPAPTPPYKPSLSNLTQDQVKQLILFHALPHYYALADFKNLSQVSPVSTFAGAGGYALNFTDVSGTVHLDSGWSKTKVSSSVHSTDPVAVYQVDKVLFLRRYLVLIYPSPAPAPAPETSLAADSPSSDSTGDGSAPGTSLQILLIGSLV
PdeFLA6	MEFSMIIMFSSTLVFLCTPVAYAQTAATPPAPTPTPSSSPAPAPAPTPPYVSLTDLLSVAGPFHTFLSYLESTKVVDTFQNQANNTDEGITIFKPSLSNLTQDQVKQLILFHALPHYYALADFKNLSQVSPVSTFAGAGGYALNFTDVSGTVHLDSGWSKTKVSSSVHSTDPVAVYQVDKVLLPEAIFGADIPPAPAPAPAPETSLAADSPSSDSTGDGSAPGTSPPNSSYRIFGVDVWSQLVLALIGVLVVFL
PdeFLA7	MISILAQERLLGAALGAAFAGFIVYEQRKRIYQSISPEHPQSQLREPIFGKQFRSEFELSWNKAVDQTFGPLVASLNSRQSPAAAPVMPPPTTPVKAPPTAPSQAPSAQVATSPGPVDVIKILQKAGHFTVFVRLMQATTEDTELNKELNKTNNGITIFAPSDSAFSSLKAGFLNALSDEDKTELVKFHVLPALISSSQFQTVSNPVRTQAGT

	GPRVTLNVTTTGNFVNITTGLTNTSISGTVYTDSQLAIYQIDKVLFPLDIFTPK<br>PPAPAPAPELGKPRKAAPGVESPTAPKDNSGALTPLILHDNALLLAVSCMVAA<br>IFS
PdeFLA8	MESSPKLSILLILSLSIIISSTSIDGVETTTFSSNLSPQSPQPQISTSDHFHDHSFSS<br>HTNLLAPILSHLGFTQLAMAVPSLPADSTTTAWSGPSTLFAPSDSSLRTCFSCS<br>IPDLLHEHIVPGLFSIDYLRKLAFGTKIETLSPGRCITVTSTSLKNDSATPSTVK<br>VFIGGVEITHPDLFNNGVLIIHGIRGYIAPLSPFSCDFERLSSLSFPFQEGVTPH<br>VTSTTHQQGIGTLVQPAIMRLMLRDAMLRLRSNGFTILSLAMRVKYPELTNL<br>VNMTVFALDDVSIFSGSHGYISSVRFHIVPNHYLSTADLERLPVGATLPTLER<br>GQALVVTSAGGLTGFNTAVPMRINYVRVKVPDVMRNLKIVVHAVYLPFPRIH<br>PTSAAAFDEMMGIGGEGQNIVAAEDAACSAVFEEDGSCGMVPPMPAQVKPS<br>VVVRSDEDHHGL
PdeFLA9	MRKQLLSPFVPFLMFFLYSSTTVAQTPSPAPSGPTNITAILEKAGQFTTLIRLM<br>KSTQEADQINTQLNNSNQGLTVFAPPDNAFTNLKAGTLNSLSDQQKVQLVQ<br>FHIIPNFFSMSSFQTVSNPLRTQAGNSADGEFPLNVTTSGNQVNITTGVNTAT<br>VANTIFTDGQLVVYQVDQVLLPLDLFGTAAAPAPAPSKPDKDVPAKAPAGSK<br>ADASVDSSESKGVDEEM
PdeFLA10	MARINPAISQITPTTTLTYFLLLLLTTTTTITPILAITNLTALLSSFPDFSSFTSLL<br>ASTPSITSDLSNRSALTLLVIPNSYLSSSLDLTRRLSPSSLTDLLRYHILLQYLSS<br>SDLHQLPPAGALITTLFQTTGRASSNFGSVNITRNPVTNAITISSPSPFSSSNAT<br>VLSLIKTLPYNVSIISVNSLLVPYGFDLMASETRPSLGLNITKALLDGRSFFVA<br>ASLLSASGVVQEFEADEGGAGITLFVPTDSAFSDLSETDVSLQSLPADKKAD<br>VLKFHVLHSYYPLGSLESIVNPVQPTLATEDMGAGSFTLNISRANGSVAIDSG<br>IVLASVTQTVFDQNPVAIFGVSKVLLPKEIFGRNPVLTSKPGNSDMGNALPPA<br>VALSPESSPKMLSSAPGVREENSGVGGLQRFSTLPLLCALVVWYCI
PdeFLA11	MDSHICGVSKKTLFLFTLLCLSVSSISALPHQNKTGNSTGTGQMINSNSVLVA<br>LLDSHYTELAELVEKALLLQTLEEAVGKHNITIFAPRNEALERQLDPEFKRFL<br>LEPGNLKSLQTLVLFHIIPQRVGSNDWPGHKSNPSRHTTLCNDHLHLITKNSG<br>KKVVGSAELTRPDDVTRPDGVIHGIEQLLVPQSVQEDFNRRRNLRSISAVLPE<br>GAPEVDPRTHRLKKPEPPVRAGSPPVLPIYDAMAPGPSLAPAPAPGPGGPHH<br>HFDGESQVKDFIQTLLLYGGYNEMADILVNLTSLATEMGRLVSEGYVLTVLA<br>PNDEAMAKLTTDQLSEPGAPEQIIYYHIIPEYQTEESMYNAVRRFGKIGYDTL<br>RLPHKVAAQEADGSVKFGSGDGSAYLFDPDIYTDGRISVQGIDGVLFPEVEK<br>ESTSVKKSVSAVKVATTKPRRGKLMEVACIMLGTLGQDSRFTTCQ
PdeFLA12	MIHQYCHCYCWLKTLSNYRKLSTLLFIPYITRPHILICRLRNSFSTMNSPSTYF<br>WSTSLHAYKGLCIISFEFKHKGSDNNNKEGFGFIFLAKMDSKASSLLFIAFLC<br>LISTSTAFNITKILAQYPEFANFNDLLSQSGLAQEMNSRQTITVLVLDNGSIDG<br>LSGRPLDIAKRILSAHVILDYYDQIKLSKLQKASTIVTTLYQASGVADNRQGF<br>LNISRTAEGIKFGSAMKGAPLVASLVKSIYSQPYNISVLQVSEPIETPGIENMAP<br>PPPPGTAAVPKKAPAPAPSTKTPPAAPPTAKTPAKSPAKSPSKAPAPSKEGPSTP<br>TEAPAEGPVAADGPVAAGGPVADVPADSPEADTEVAEEAPAVAPAKAASSRM<br>HVAGATVVIGLFACIMGF
PdeFLA13	MAPSPPCIHIFFASILLLSNFHLGFSSSSSSTLQENHSNGSYSGQINSNSVLVA<br>LLDSHYTELAELVEKALLLQTLEDAVGKHNITIFAPRNEALERDLDPEFKRIN

	LSHNSSLHHHSTLCRDRIKLGSQSGEKLIDSAKIIQVNAVERPDGVIHGIERLL<br>IPRSVQQDFNNRRSLQSISAVKPEGAPEVDPRTHRLKKPAPPAKPGSAPVLPIY<br>DAMAPGPSLAPAPAPGPGGPHHHFNGERQVKDFIETLLLYGGYNEMADILV<br>NLTSLATEMGRLVSEGYVLTVLAPNDEAMAKLTTDQLSEPGAPEQIIYYHVIP<br>EYQTEESMYNAVRRFGKISYDTLRLPHKVLAQEADGSVKFGHAENSAYLFD<br>PDIYTDGRISVQGIDGVLFPLEEKEKSDTKTEMKSVKVAAKPQRRGKLLEVA<br>CRMLGTFGQDSHFTTCQ
PdeFLA14	MYFFYSVQHRPHLCPTMQPFILLFWLLFLHTCSQTFCQSPAQSPAATQTKAP<br>VSPPPPAGPTDTIQILLKAGRFLSFVRLMKATHVDTQLFSQLNSSTDGITMFAP<br>NDNAFTSLVAGAVGSLNDREKLEFVQFHILPRFLSISDFQTLSNPVKTLAGSD<br>RKFPLTITTSDNSVTVSSGLTKTSLSNTIYTDKQVAIYEINKVLVPRDLFPPAPP<br>APAPAKPLAEPDPVAPRDASSALVIAWQHRVNVVLFGAGLYIAALVMDP
PdeFLA15	MRQRSFVLVLSLVFFFLHCTKTLCQSPAAAPAMTPPKTPVKAPPADSSQAPSA<br>QVATSPGPVDVNKILQKAGHFTVFARLMQATTEDTELNKELNTTNNGITILA<br>PTDNAFSSLKAGFLNSLSDEDKTELVKFHVLPAFISTSQFQTVSNPVRTQAGT<br>GPRVTLNVTTTGNFVNISSGLTNTSISGTVYTDSQLAIYQLDKVLFPLDIFTPK<br>PPAPAPEPALGKPKKAAPDAESPSAPKDISGAPALLFLLNNALVLAVSCAFGA<br>MIYS
PdeFLA16	MAIALSSFNIFFTFLLVSTFHLGFSFSALQENHSNGTYSGQINSNSVLVALLDS<br>HYTELSELVEKALLLQTLEDAVGKHNITIFAPKNEALERDLDPEFKRFLLEPG<br>NLKSLQTLLLYHIVPNRINPSHNSSLQHHSTLCRDRVKLSSQESGEKLIDSAKI<br>IQVNAVERPDGVIHGIERLLIPRSVQQDFNNRRSLQSISAVKPEGAPEVDPRTH<br>RLKKPAPPAKPGSAPVLPIYDAMAPGPSLAPAPAPGPGGPHHHFNGEKQVKD<br>FIETLLLYGGYNEMADILVNLTSLATEMGRLVSEGYVLTVLAPNDEAMAKLT<br>TDQLSEPGAPEQIIYYHVIPEYQTEESMYNAVRRFGKISYDTLRLPHKVLAEE<br>ADGSVKFGHPENSAYLFDPDIYTDGRISVQGIDGVLFPLEEKEKSGPKRKLR<br>ALRLLLSHKGEGGCLKWLAGCLGHLDKIHISPHASK
PdeFLA17	MQRLTILLSLLFLLSTSTTFTRGHNITHILGKHPSFSTFNHYLTLTHLAGEINSR<br>TTITVCAVDNAAMSELLSKHPSIATIKNILSLHVLLDYFGTKKLHQIREGTAL<br>AATMFQATGSAPGSTGFVNITDVKGGKVAFGPEDNEGNLDVFYVKSLEEIPY<br>NISVIQISKVLPSDVAAAPTPEPSAMNITDIMSAHGCKVFAGTLIANPEASKTY<br>QDNIDGGLTVFCPLDDPFKAFFPKFKNLTASGKVSFLEFFGVPIYQSLAMLKS<br>NNGIMNTLATDGEKKFDFTVQNDGEDVTLKTRSITAKIVGTLIDEQPLAIYTI<br>DKVLLPKELFKAAPTPAPAPAPEKEVADAPKSSKHKKPSSDVVPSDSPADSPD<br>GDLADQTADDNASVTLYGGRLVAMVLSLCSGLLLL
PdeFLA18	MKHHFSVFLFPAIFLLLHCTQTLSQTPTAAPAKAPAAASAPPPAATSSAQASPP<br>VMVPVQVSKGPVNVIKILQKAGGFAVFIRLIKSTQEDIQVFSQLNDSRDGVTI<br>FAPTDGAFSAIIKSGSGSRFALNVITTENMVNVTSGLTNTSVSAIVYTDSQLAI<br>YQVDKVLLPLDIFAPKALAPAPAPPKPKKDDGAESPLVPEDTSSSVSCIPLNSL<br>IIFGAGMVAAVFTL
PdeFLA19	MVPQFLLSFSLILSFLLHCPPTLAQSPAAAPGPPGPTNVTKILEKGGQFSVFIR<br>LLKATQEDVTLNGQLNNTNNAITIFAPSDNAFSSLKSGTLNSLNDQEKAELV<br>QFHIIPQYLSSSQFQTVSNPLTTQAGSGGRLELNVTTTGNSVNITTGLTNTSVS<br>GTIYTDNQLAVYQVDKVLLPVDIFTPKPPTPAPAPEKPKKRSKAAESPDAPED

	NSGAVSLTVLNDVVFFGVGIVAAIFSL
PdeFLA20	MKQQYYSLFSFSFFLLFLHCTTTFAQTSPAATPAQAPAVVAAQPHGITNVTKIL<br>EKAGHFTIFIRLLRSTQEENHLFSALNDSSSGVTIFAPTDSAFSELKSGTLNTLS<br>DGDKSELVKFHVVPTFLSTSQFQTVSNPLGTWAGTGNRLPLNVTSYPNSVNI<br>TTGLTNTSLSGTVYTDNQLAIYKIEKVLLPKDIFASKAPAPAPVAPAPEKSTKA<br>VPAATAESPVAPVDTSSALMFTQNHVVGSVAIFAAAMFAL
PdeFLA21	MATTPLSFFLLSLLLSLSLSLNAQAQTPTAPAPTPSGPVNFTAVLVKGGQFATL<br>IRLLNSTQTLNQIENQLNSSSEGMTIFAPTDNAFNNLKAGALNGLNQQEQVQ<br>LLQYHTLPKFYTMSNLLLVSNPVPTQASGQDGVWGLNFTGQSNQVNVSTGL<br>VEVQINNALRQDSPLAVYPVDKVLLPEALFGVKPPTASPPAPSSKSNSTVAAA<br>EPSTSKNSAGGRNVALGLVVGLGLVCMGILS
PdeFLA22	MKQQHSLSSFSFFLLLLHCANTFAQSPAATPAQAPAAVVAQPPAATPTQAAQP<br>HGITNVTKILEKAGHFTIFIRLLRSTQEENHLFSALNDSSSGVTIFAPTDSAFSE<br>LKSGTLNTLSDGDKSELVKFHVVPTFLSTSQFQTVSNPLGTWAGTGSRLPLN<br>VTSYPNSVNITTGLTNTSLSGTVYTDNQLAIYKIEKVLLPKDIFASKAPAPAPV<br>APAPEKPTKAVPSANVESPVAPVDISSAVTFMHNNVVGSLVIVAAAMFACNV<br>EGF
PdeFLA23	MASCSHWWHAPVYFIASAVLAFIAISTAMNSPSNNATRPTRPTSNYLSLNAS<br>RTLRESGFNIMATLLLISPEMFFLSPNTTIFAIKDSSLVNTSLPPWFLKNLLQYH<br>TSPLKLSMEDVFKKPQGSCFPTLVDRKKLAVTKIDAKERLAEINHVLVSHPD<br>MVLERRITIHGVLAPFSSLRSKDVYFGWESIQAPICDANSSLVSDANGPRIILE<br>WTRIIHLLSSHRFVSFAIGLNSVLDRILADHKNLSSVTIFAPPELEFVASSSPML<br>EKIVRLHILPQRATYIELAALPDKQRLRTLLPDEDLKITKGVGVTQGLAINGV<br>EIAAPEIFSSKEFIVHGITQAFKMAKFPNASR
PdeFLA24	MATLQYSLLLSFTLSALVSTILAHNITDILSGFPEYSEFNKYLTQTKLADEINT<br>RQTITVLALNNGAMTALAAKHPLSVIKNALSLLVVLDYYDPTKLHQISKGTT<br>LSTTLYQTTGNAPGNLGFVNITDLQGGKVGFGSAAPGSKLDSSYTKSVKQV<br>PYNISILEISQPIIAPGILTAPAPTPSSVNITALLEKAGCKTFASLLQTSGVIKTYQ<br>SAADKGLTIFAPNDEAFKAAGVPDLSKLTNAEIVSLLQYHATATYSPFGSLKT<br>SKDPISTLASNGAGKFDLTVTSAGDSVTLHTGIGPSRVAETVLDSTPLVIFTVD<br>NVLLPVELFGKAPSPAPAGEPVSAPSPSPSPVASSPAPASVEAPSPLAASPPAPP<br>VETPGGAPADAPFGSENSTADGSAAVHVSVPVQVTVFATVICSILMS
PdeFLA25	MKFSMIIVLSSTLLFSCTPLAYAQKVASPPAPTPTPSPAPAPSPPYVNLTDLLSV<br>AGPFHNFLNYLESTKVIDTFQNQANNTDEGITIFVPKDDAFKNLKKASLSNL<br>TQDQLKQLILFHALPHYYSLSDFKNLSHVSPVSTFAGAGGYALNFTDTSGTV<br>HLDSGWSKTKVSSSVHSTDPVAIYQVDKVLLPEAIFGTDIPPTPAPAPAPDTSP<br>TADSPTSDDSAGAGSAPGKSPPNSSYRINGVGIWSQLVLAIAGVLVLFL
PdeFLA26	MPRSLPLLALAISLVLIASTTTVNAHNITRILAKHPQFSTFNHYLTVTHLAAEI<br>NRRQTITVLALDNAAMSSLISKQLSVYTLRNVLSLHVLVDYFGTRKLHQITN<br>GTELTATMFQATGSAPGASGYVNITDLNGGKVAFGAEDNDGKLDAVYVKSL<br>EEIPYNISILQISQPLNSAEAEAPTAAPTLNVTAILSNQGCKAFSDLLIASGAHT<br>TFEENVDGGLTVFCPTDPVINGFMPKYKNLTAPQKVSLLLYHGIPIYQSLQML<br>KTSNGIMNTLATNGANKYDFTVQNDGEVVTLETKEPLVVYKINKVCCPGSC<br>LRRLRRKKHRRLRGEGCADGPNADAPSDESDDQTADNDNGVNKMGGGRL

	AVVARVSFLGW
PdeFLA27	MVPQFLFSASFILFFLLHCPPTLAQSPAAAPAPPGPTNVTKVLEKGGQFSVFIR<br>LLKATQEDVTLNGQLNNTNNAITIFAPSDNAFSSLKSGTLNSLSDQEKAELVQ<br>FHIIPQFLSSSQFQTVSNPLTTQAGSGGRLELNVTTTGNSVNITTGLTNTSVSG<br>TIYTDNQLAVYQVDKVLLPLDIFTPKPPTPAPAPEKSKKRSKAAASPESPADT<br>SGAVSFTVLNNVVFFGVCMVAAIYSL
PdeFLA28	MDSHIYDVSEKTLFLFTLLCFSVASISALPHQNRTGNSTVAGQMINSNSVLVA<br>LLDSHYTELAELVEKALLLQTLEEAVGKHNITIFAPKNEALERQLDPEFKRFIL<br>EPGNLKSLQTLLLFHIIPQRVGSNDWPGHKSNPTRHATLCNDHLHLITKNSG<br>KKFVGAAVLTRPDDVTRPDGVIHGIERLLVPQSVQEDFNRRRNLRSISAVLPE<br>GAPEVDPRTHRLKKPEPPVRAGSPPVLPVYDAMSPGPSLAPASAPGPGGPHN<br>HFDGESQVKDFIQTLLQYGGYNEMADILVNLTSLATEMGRLVSEGYVLTVLA<br>PNDEAMAKLTTDQLSEPGAPEQIIYYHIIPEYQTEESMYNAVRRFGKIGYDTL<br>RLPHKVVAQEADGSVKFGSGDGSAYLFDPDIYTDGRISVQGIDGVLFPEVEK<br>ESTSVKKSVSSVKVATTTPRRGKLMEVACRMLGSLGQDSHFTTCQ
PdeFLA29	MRKQLLSPFVPFLMFFLYSSTTFAQTPSPAPSGPTNITAILAKAGQFTTLIRLLK<br>STQEADQINTQLNNSNQGLTVFAPADNSFANLKAGTLNSLSDQQKVQLVQF<br>HILPNFLSMSNFQTVSNPLRTQAGNSADGEFPLNVTTSGNQVNITTGVNTATV<br>ANTIYTDGQLVVYQVDQVLLPLDLFGTAPAPAPAPSKPEKDVPAKAPAGSKE<br>DASVDSSGASIATVSFGIVLIAAISLKL
PdeFLA30	MSTMLLFLLILLLISSSVLAASSPFSNAMEILSTSGYLSMALTLEITSKRLHLES<br>SAATIFAPLDIALARLGQLSVLDLQYHISPVRLSGYYLDSLPFGTRIPTLLPNH<br>SLIVTTSLSYFDGKLSINGISIEESALVDFGSLIIFGTSEFFNSSLEISPNLTPAPAP<br>SPSPAWMSTFFGQASHLLMPRGYSIMGTFLDAQLFGIKNQTRLTIFAPVDQA<br>MDAYAKNVSDYSSIFRKHVVPGLFPRQDLEGFNDGTSLPTFSGGFMINLTKS<br>GDVLVLNSVPVIFPEMYQSDWLIIHGLNQLLMPPLKEEELVGESFSELDGAE<br>DKPDVLDFDDYVYGAP
PdeFLA31	MAPINPAISHITPTTTLTYFLLLLLATTTTTTPILAITNLTALLSSFPDFSSFTSLIT<br>SIPSLTSDLSDRSALTLLVIPNSYLSSSLDLTRRLSPSALADLLRYHILLQYLSSS<br>DLHQVPPSGALITTLFQTTGRASSNSGSVNITRNPVTNAITISSPSPFSSSNATV<br>LSLIMTLPYNVSIISVNSLLVPYGFDLMASETRPTLGLNITKALLDGHSFLVAA<br>SLLSASGVVQEFEADEGGAGITLFVPTDSAFSDLSATAISLQSLPADKKADVL<br>KFHVLHSYYPLGSLESIVNPVQPTLATEDMGAGSFTLNISRVNGSVAIDSGIV<br>QASVTQTVFDQNPVVIFGVSKVLLPKEIFGRNPMLTSKPGNTDMGNAQPPVS<br>ALSPENSPKMLSSAPGVREEIKSGVGGLQWLSTLHLLCVVFVCNCI
PdeFLA32	MGPQNLMINKSTAKILLHLLLLSLLHQITTATLTDQELDFALSSLRSYGYTLFP<br>NAISTSDLRLQLLNQSSNATSTSTFTLFCPPDSLLFSVDLASTAPHYTKSLFLH<br>VSPSRLSTSDLRNLTAASGGTYIDSLVPNHRLLIANSLAQLNGTVDGSILVNR<br>VRVSVPDLFLGSDIAVHGLDGILVAGFEDKVEDTSFEAATWSPANAIGSAERN<br>SPLAGRFPARRRKGRNHRQNGRNGGIRRNHRGRRINGGRRHRGGRNVSGG<br>TRGDGVTHGAFAMYNHRL
PdeFLA33	MEAFTTLLVLLMIKVLVCSTSPTDIPSRSQDLFVATDEMARANYFSFVMLINM<br>APLDQKFQGNVTFLMPKDRILSKIRMHPNAVSDFLLRHSIPSPLLFDHLLHIPP<br>GSLIPSSNPDYMLNISNKGRKSFFLNNVKISSPDLCTAGSSIRCHGIDGVLLVA

	TDRHPLPACSNSTSPAVVATPPSPSLPLPDIPSFPSSAPPPGAAAPTDQEHNPKH<br>SGSSQLESLSLGGLLKFMATSILLLNARVLYTVGLN
PdeFLA34	MATSPLSLVLLSVFLSLSLHAQAQAPAAPAPAPSGPVNFTAVLVKGGQFVTFIS<br>LLNKTQTFNQIENQINSSSEGMTIFAPTDNAFSNLKSGALNGLSQQQQVQLL<br>QYHMLPKFYSLSNLLLVSNPVPTQASGQEGVWGLNFTGQSNQVNVSTGLVE<br>VQVNNALRQDFPLAVYPVDKVLLPEELFGAKPPSASPPAPATKGSSSGKSNSS<br>DTAAEPSPGKNSAGGRNVALGLIFGLGFVSVGILS
PdeFLA35	MKQQSISFFIFLLFLQCTYTFAQSPAAAPAQAPAVVASPPAATPTQAAAPHGIT<br>NVTKILEKAGHFTIFIRLLRSTQEENHLFSALNDSSTGLTIFAPTDSAFSELKSG<br>TLNTLSDGDKSELVKFHVVPTFLSTSQFQTVSNPLGTWAGTGNRLPLNVTSY<br>PNSVNITTGLTNTSLSGTVYTDNQLAIYKIEKVLLPKDIFASNAPAPAPVAPAP<br>EKPAKAVPAANVESPVAPVDISSAVWFMHNNVVGSVGIVASAVFAL
PdeFLA36	MKQQLISSFSIFLLFLHCANTFAQIPAAAPAQAPAVVVAPPPAATPTQAAAPHG<br>ITNVTKILEKAGHFTIFIRLLRSTQEESHLFSALNDSSTGLTIFAPTDSAFSELKS<br>GTLNTLRDGDKSELVKFHVVPTFLSTSQFQTVSNPLGTWAGTGSRLPLNVTS<br>YPNSVNITTGLTNTSLSGTVYTDNQLAIYKIEKGMKKVRVSFFKAKGPKA
PdeFLA37	MASHNVTKILEKAGHFTIFIRLLRSTQEENHLFSALNDSSTGLTIFAPTDSAFS<br>ELKSGTLNTLSDGDKSELVKFHVVPTFLSTSQFQTVSNPLGTWAGTGNRLPL<br>NVTSYPNSVNITTGLTNTSLSGTVYTDNQLAIYKIEKVLLPKDIFASNAPAPAP<br>VAPAPEKPAKAVPAANVESPVAPVDISSAVWFMHNNAVGSVGIVAAAVFAL
PdeFLA38	MKQQSISSFFIFLFFLQCTYTFAQSPAAAPAQAPAVVVAQPPAATPAQAAAPH<br>GITNVTKILEKAGHFTIFIRLLRSTQEENHLFSALNDSSTGLTIFAPTDSAFSEL<br>KSGTLNTLSDGDKSELVKFHVVPTFLSTSQFQTVSNPLGTWAGTGSRLPLNV<br>TSYPNSVNITTGLTNTSLSGTVYTDNQLAIYKIEKVLLPKDIFASNAPAPAPVA<br>PAPEKPAKAVPAANVESPVAPVDISSAVWFMHNNVVGSVGIVASAVFAL
PdeFLA39	MPRSLPLLALAISLVLIASTTTVNAHNITRILAKHPQFSTFNHYLTVTHLAAEI<br>NRRQTITVLALDNAAMSSLISKQLSVYTLRNVLSLHVLVDYFGTRKLHQITN<br>GTELTATMFQATGSAPGASGYVNITDLNGGKVAFGAEDNDGKLDAVYVKSL<br>EEIPYNISILQISQPLNSAEAEAPTAAPTLNVTAILSNQGCKAFSDLLIASGAHT<br>TFEENVDGGLTVFCPTDPVINGFMPKYKNLTAPQKVSLLLYHGIPIYQSLQML<br>KTSNGIMNTLATNGANKYDFTVQNDGEVVTLETKVTTATITGTVKDEEPLV<br>VYKINKVLLPRELFKAAPEKKAPAPKGEKDVADGPNADAPSDESDDQTADN<br>DNGVNKMGGGRLAVVAPSFFFGVVMFFLFD
PdeFLA40	MRQQYVFTTLTLLILFSLSCSTTLAQAPALAPAPSGPTNVTKILEKAGQFTLFI<br>RLLKSTQVANQLLGQLNNSNNGMTVFAPTDNAFSSLKSGTLNSLTDEQKVE<br>LVQFHIVPTYLTSSQFQTISNPLRTQAGDSGDGKFPLNITTSGNSVNITTGLTN<br>TSVSGTIYTDGQLAVYQIDQVLQPLQIFAPRPPAPAPAPAKSKNKKATTVADSP<br>DVTPADNSKAATLQNVGLFGVAALVIALSL

Green: Signal peptide; Red:AGP-like glycosylated regions;
Yellow: Fasciclin domain; Gray: GPI-anchor protein;
