# Supplementary material for: Genome-Wide Comparative Analysis of the Fasciclin-like Arabinogalactan Proteins (FLAs) in Salicacea and Identification of Secondary Tissue Development-Related Genes
Source: Int J Mol Sci. 2023 Jan 12;24(2):1481. doi: 10.3390/ijms24021481 (PMC9862198; doi:10.3390/ijms24021481)
Supplement: Supplementary file 1 [file ijms-24-01481-s001.zip › Supplemental Table S2.pdf]

Supplemental Table S2. Prediction of conserved structural domains of FLA proteins in *Salix suchowensis.*

Name	Precursor protein backbones
SsuFLA1	MDSHIYGVSKQTLFLFTLLCFSVASISALPHQNKTGNSTVTGQMINSNSVLVA<br>LLDSHYTELAELVEKALLLQTLEEAVGKHNITIFAPRNEALERQLNPEFKRFL<br>LEPGNLKSLQTLLLFHIIPQRVDPAHHSLQRPPGVNHKNSGKKLVGSAALTRP<br>NDVTRPDGVIHGIERLLVPQSVQEEFNRRRNLRSISAVLPEGAPEVDPRTHRL<br>KKSEPPVRAGSQPVLPIYDAMSPGPSLAPAPAPGPGGPHHHFDGESQVKDFI<br>QTLLHYGGYNEMADILVNLTSLATEMGRLVSEGYVLTVLAPNDEAMAKLTT<br>DQLSEPGAPEQIIYYHIIPEYQTEESMYNAVRRFGKIGYDTLRLPHKVVAQEA<br>DGSVKFGSGDGSAYLFDPDIYTDGRISVQGIDGVLFPEVEKESTSVKKSVSSV<br>KIATTTPRRGKLMEVACRVLGTFGQDSHFTTCQ
SsuFLA2	MRKQLLSPFVPFLMFFFLYSSTSLAQTPSPAPSGPTNITAILEKAGQFTTLIRL<br>MKSTQEADQINTQLNNSNQGLTVFAPTDNSFSSLKAGTLNSLSDQQKVQLV<br>QFHILPSFLSMSNFQTVSNPLRTQAGNSADGEFPLNVTTSGNQVNITTGVNSA<br>TVANTIYTDGQLVVYQVDQVLLPLDLFGAAPAPAPAPSKPEKDVPAKAPAGS<br>KEDASVDASGASIAAVSFGAVLIAAISLKI
SsuFLA3	MSEMLSKNPSITTIKNILSLHVLLDYFGAKKLHQIRDGSALSATMFQATGSAP<br>GSTGFVNITDVKGGKVAFGPKDNGGNLDVFYVKSVEEIPYNISVIQISKLFPS<br>DVAAAPTPAPSAMNLTDIMSAHGCKVFADTLIANPDASKTYQDNIDGGLTVF<br>CPLDDPFKAFLPKFKNLTASGKESFLEFYGVPIYQSLAMLKSNNGIMNTLAT<br>NGDKKFDFTVQNDAEDVTLKTRSTTAKIVGTLIDEQPLAIYSIDKVLLPKELF<br>KAAPTPAPAPAPEEAADAPKSSKHKKPAADAAPSDSPADSPEGDAADQTADN<br>NASVRLDGGRLVAIVLSLCLGLLLL
SsuFLA4	MDKYLLSFLLLLTTTTTTATSRSSTFNIISTFGPTPSPTSAATPAPNPITNSNTRI<br>AFSPTYAPSPITTVPTTPAPSSAPTPINNTTNTSATLAPTTTPLTAPAPTPATETTL<br>TPTSAPSPAPTPTPTTETTLTPTSAPITAPTPTSITSILETSPAPTPISTPVTTVTFS<br>LSPTPRSPLTPQQDELKFVIQEQIYNVIDAILGAGDFKNWANALTMADSTTFPI<br>SSTFFIPSDNSLSPATTTADPDIVPYHIVPQRLSFADLQQFKAFSRLPTLLFDKSI<br>LITNNSASNFTLDASRLTHPNIYTNAAIAVHGIDNLLDHSVYGTESGKKSSKP<br>NAAGPPPTPDSPPPRAFVPSTADHEELTVHHHGESDAACLCTGVWTVFLVLC<br>AALASKFHIMTLVH
SsuFLA5	MQQFILLLSLLFLHNCSRTLCQSSVQSPTPTKAPVPPPSLAVPTDTIKILLKAG<br>RFLSFVRLMKATQVDTQLFSQLNSSTDGITIFAPNDNAFSSRVAGAVGSLNDR<br>EKLEFVQFHILPRFLSISDFQTVSNPVKTLAGSDNKLPLTITTSDNSVKISSGLT<br>KTSISNTIYTDKQVAIYEVDKVLVPKDLFPPAPPAPAPSKPVEESPVVPRDASA<br>GAIIVLHHHLVFFGVGVYIAALVFTL
SsuFLA6	MRQRSVSLVLFSLILFFLQYCTRTSGQSPAAAPAMPPPTTPVKAPPNAPSQAP<br>SAQVATSPGPVDVGKILQKAGHFTVFVRLMQATTEDAELNKELNTTNNGITI<br>FAPSDNAFSSLKSGFLNALSDEDKTELVKFHVLPALISSSQFQTVSNPVRTQA<br>GTGPRVALNVTTTGNFVNISTGLTNTSISGTVYTDSQLAIYQIDKVLFPVDIFT<br>PKPPAPAPAPELGKPGKAAPGAESPTAPKEISGALTPLILVHNNALTLAVSCMV

	AAIFS
SsuFLA7	MTMRKQLLSPLVPFLIFFLCISTTVAQTPSPAPSGPANITAVLEKAGQFATFIRL MKSTQEANQINTQLNNSNQGLTVFAPTDNAFTNLKAGTLNSLSDQQKVQLV QFHILPNFLSMSSFQTVSNPLRTQAGNSAAGEFPLNVTTSGNQVNISTGVDTA TVANTIFTNGQLVVYQVDQVLLPLDLFGTAAAPAPAPSKPDKDVPAKAPAGS KEDASVDTSGATIAAVSVSAVLIAAISLKL
SsuFLA8	MRRQSFIAFSLFFFLLCTKTLGQSPAAAPVKAPPAAAAQTPAAQVAASPGPV DIIKILQKAGHFTVFVRLMQATTEDIELNKELNTTNNGITIFAPSDNAFSSLKA GFLNSLSDEDKTELVKFHVLPAFIASSQFQTVSNPVRTQAGTGPRVTLNVTTA GNFVNISTGLTNTSLSSTVYTDSQLAIYQVDKVLFPLDIFTPKPPAPAPAPELE RPKKAAPDVESPTAAKDISGAFTAVVHNNAVLLAISVVAAIFFP
SsuFLA9	MGMAQINPAISHITPTTTLCYFLLLLLSTTPILAITNITALLSSFPDFSSFTSLLA STPSITSDLSNRSALTLLVIPNSYLSSSLDLTRRLSPSSRTDLLRYHILLQYLSSS DLHHLPPAGALITTLFQTTGRASANFGSVNITRNPVTNAITISSPSPFSSSNATV LSLIKTLPYNVSIISVNSLLVPYGFDLMASETRPPLGLNITKALLDGHNFFVAA SLLSASGVVQEFEAYEGGAGITLFVPTDSAFADLSENDPTLATEDMGAGSFT LNISRANGSVAIDSGIVLASVTQTVFDQNPVAIFGVSKVLLPKEIFGSNPVLTS KPGNSDMGNAQPPAVALSPESSPKMLSSAPGAREEIKSGVGGSQWLSNLPFL CVVAVWYCI
SsuFLA10	MDSHIYGVSKKTLFLFTLLCLSVSSISALPHQNKAGNSTGSGQMINSNSVLVA LLDSHYTELAELVEKALLLQTLEEAVGKHNITIFAPRNEALERQLDPEFKRFL LEPGHKSNPSRHTTLCNDHQHLITQNSGKKLVGSAEVTRPDDVIRPDGVIHGI ERLLIPQSVQEDFNRRRNLRSISAVLPEGAPEVDPRTHRLKKPEPPVRAGSPP VLPIYDAMSPGPSLAPAPAPGPGGPHHHFDGESQVKDFIQTLLLYGGYNEMA DILVNLTSLATEMGRLVSEGYVLTVLAPNDEAMAKLTTDQLSEPGAPEQIIYY HIIPEYQTEESMYNAVRRFGKIGYDTLRLPHKVVAQEADGSVKFGSGDGSAY LFDPDIYTDGRISVQGIDGVLFPEVEKESTSVKKSFSSVKVATTNPRRGKLME VACRMLGTLGQDSRSLHVNEYTVKSRRKRKKKRVKMAGTMKTIDKW
SsuFLA11	MDSHIYGVSKKTLFLFTLLCLSVSSISALPHQNKAGNSTGSGQMINSNSVLVA LLDSHYTELAELVEKALLLQTLEEAVGKHNITIFAPRNEALERQLDPEFKRFL LEPGNLKSLQTLLLFHIIPQRVGSNDWPGHKSNPSRHTTLCNDHQHLITQNSG KKLVGSAEVTRPDDVIRPDGVIHGIERLLIPQSVQEDFNRRRNLRSISAVLPEG APEVDPRTHRLKKPEPPVRAGSPPVLPIYDAMSPGPSLAPAPAPGPGGPHHHF DGESQVKDFIQTLLLYGGYNEMADILVNLTSLATEMGRLVSEGYVLTVLAPN DEAMAKLTTDQLSEPGAPEQIIYYHIIPEYQTEESMYNAVRRFGKIGYDTLRL PHKVVAQEADGSVKFGSGDGSAYLFDPDIYTDGRISVQGIDGFCFPRSRKRV LLLRNPSVLLRLPLPIQEEGN
SsuFLA12	MDSHIYGVSKKTLFLFTLLCLSVSSISALPHQNKAGNSTGSGQMINSNSVLVA LLDSHYTELAELVEKALLLQTLEEAVGKHNITIFAPRNEALERQLDPEFKRFL LEPGNLKSLQTLLLFHIIPQRVGSNDWPGHKSNPSRHTTLCNDHQHLITQNSG KKLVGSAEVTRPDDVIRPDGVIHGIERLLIPQSVQEDFNRRRNLRSISAVLPEG APEVDPRTHRLKKPEPPVRAGSPPVLPIYDAMSPGPSLAPPSSRTRWAHHHFD GESQVKDFIQTLLLYGGYNEMADILVNLTSLATEMGRLVSEGYVLTVLAPND EAMAKLTTDQLSEPGAPEQIIYYHIIPEYQTEESMYNAVRRFGKIGYDTLRLP

	HKVVAQEADGSVKFGSGDGSAYLFDPDIYTDGRISVQGIDGVLFPEVEKEST SVKKSFSSVKVATTNPRRGKLMEVACRMLGTLGQDSRFTTCQ
SsuFLA13	MDSKASSLLFIAFLCLISTSTAFNITRILAQYPEFASFNDLLSQSGLAQEMNGR QTITVLALDNGSIDGLSGRPLDIAKRILSAHVILDYYDQIKLSKLQKASTIVTT LYQASGVAEDRQGFLNISRTAEGIKFGSAMKGAPLSASLVKSIYSQPYNISVL QVSEPIETPGIENMAPPPPPGTAAVPKKTPAPAPSTKTPPAAPPTAKTPAKSPAK SPSKAPAPSKEGPSTPTEAPAGGPVADGPVADVPSNSPGPDKAASSRMHVAG ATVVIGLFACIMGF
SsuFLA14	MESSLKFLILLIFSFSIITISIAFDGVETTTNLSPSPPPQQQRITSPPPQVSNSDNL HDHSFFSHTTLLAPILSHLGFTQLAMVAPSLSADPTTTAWSGPFTLFAPSDSSL STCFSCSIPDLLREHIVPGLFTIDYLRKLAFGTKIETLSPGRCMTVTSNSLNND TVSHSSVKVFIGGVEITHPDLFNNGVLIIHGIQGYIAPLSPLSCDVERLSSLSFP NHERVTPHVTSTTHQQGVGTLMQPAIMRLMLRDAMLRLRNNGFTILSLAMR VKYPELVTLANMTVFALDDVSIFSGSHGYINSVRFHIVPNHYLSITDLERLPV GASLPTLERGQALVVTSAGGFNMAVPMRINYVRVKVPDLMKNLKIAVHSVY LPFPRIHPTSAAPYDEITGGGGGGYNVVAAAEAAEDGACSMVYEEDGSNCW MVPPMPAQVKPSVAVQIDEDHHGL
SsuFLA15	MESSLKFLILLIFSFSIITISIAFDGVETTTNLSPSPPPQQQRITSPPPQVSNSDNL HDHSFFSHTTLLAPILSHLGFTQLAMVAPSLSADPTTTAWSGPFTLFAPSDSSL STCFSCSIPDLLREHIVPGLFTIDYLRKLAFGTKIETLSPGRCMTVTSNSLNND TVSHSSVKVFIGGVEITHPDLFNNGVLIIHGIQGYIAPLSPLSCDVERLSSLSFP NHERVTPHVTSTTHQQGVGTLMQPAIMRLMLRDAMLRLRNNGFTILSLAMR VKYPELVTLANMTVFALDDVSIFSGSHGYINSVRFHIVPNHYLSITDLERLPV GASLPTLERGQALVVTSAGGFNMAVPMRINYVRVKVPDLMKNLKIAVHSVY LPFPRIHPTSAAPYDEIIGGGGGGYNVVAAAEAAEDGACSMVYEEDGSNCW MVPPMPAQVKPSVAVQIDEDHHGL
SsuFLA16	MAMAPSTSCSNIFFAFLLLLSFFYPGFSSSPSTLQENHGNGSYSGQISSNSVLV ALLDSHYTELAELVEKALLLQTLEDAVGEHNITIFAPKNEALERDLDPEFKRF LLEPGNLKSLQTLLLYHIVPNRINLSRNTSAHHRSTLCRDRIKLSSQESGEKFI DSAKIIQVNAVERPDGVIHGIERSLQSISAVKPEGAPEVDPRTHRLKKPAPPSK PAPAPGPGGPHHHFNGEKQVKDFIETLLLYGGYNEMADILVNLTSLATEMGR LVSEGYVLTVLAPNDEAMAKLTTDQLSEPGAPEQIIYYHVIPEYQTEESMYN AVRRFGKISYDTLRLPHKVLAQEADGSVKFGHAENSAYLFDPDIYTDGRISV QGIDGVLFPLEEEKLDTKTEIKSVKVAAKPQRRGKLLEVACRMLGTFGKDSH FTTCQ
SsuFLA17	MAATKILLSLLSLFSFLPSSFSIPTETLREAAVTLSNSGYLSMSLTLPLVSNSLL PHTPSLTIFSPCDTAFTQAGQPPLSILRLHFSPLSFPLNSLESLSLGAKIPTLFPN HSLTITSTGYDISLNGVKIRDSPVYDDGSLVILGVDRFFDPGFGASGQNLGCS VKVSSDCYSYNEASRVLKSKGYSVMASFLDLQLMAGFKDKTALTIFAPVDE VIKAFLGDLSEYSSMFRKHVVPCKIMWGDLVNFDDGVVLETYLKGFGIAVS TSGDNLMLNDQASVNFPDMYHNDWLVIHGLRSILMEPESEYSFLDDGDEF
SsuFLA18	MRPQSFISVLSLILFFLHCTKTLCQSPAAAPAMAPPTTPVKAPPADSSQAPSAQ VATSPGPVDINKILQKAGHFTVFARLMQATTEDTELNKELNNTNNGITILAPT DSAFSSLKAGFLNSLSDEDKTELVKFHVLPAFISTSQFQTVSNPVRTQAGTGP

	RVTLNVTTTGNFVNISSGLTNTSISGTVYTDSQLAIYQLDKVLYPLDIFTPKPPAPAPEPALGKPGKAAPGVESPTAPKDISAAPTQLFLHDNALLLAVSCAFAVIIYS
SsuFLA19	MAMAMAQSSSNIFFTFLLFSTFHLGFSSSALQENHSNETYSGQINSNSILVALLDSHYTELAELVEKALLLQTLEDAVGKHNITIFAPKNEALERDLDPEFKRFLLEPGNLKSLQTLLLYHIVPDRVNLSHTSSVHHHSTLCSDRVKLSGQKSGDNLIDSAKIIQVNAVERSDGVIHGIEKLLIPRSVQQDFNNRRSLRSISAVKPEGAPEVDPRTHRLKIPAPPEKPGSPPVLPIYDAMAPGPSLAPASTPAPGGPHNHFNGEKQVKDFIETLLSYGGYNEMADILVNLTSLATEMGRLVSEGYVLTVLAPNDEAMAKLPTDQLSEPGAPEQIIYYHVIPEYQTEESMYNAVRRFGKISYDTLSLPHKVLSQEADGSVKFGDAENSAYLFDPDIYTDGRISVQGIDGVLFPIEEKEKSQTKMEIKSVKVAVKPQRRGRLLEVACRMLGTFGQDSHFTTCQ
SsuFLA20	MQRLTLLLSLIFFLSTATTFTRGHNITHILGKHPSFSTFNHYLTLTHLAGEINRRTTITICAVDNAAMSELLSKHPSIATIKNILSLHVLLDYFGTKKLHQIRDGTALAATMFQATGSAPGSSGFVNITDVKGGKVGFGPEDNEGNLDVFYVKSLEEIPYNISVIQISKVLPSDVAAAPTPEPSAMNLTDIMSAHGCKVFADALLANPEASKTYQDNMDGGLTVFCPLDDPFKAFSPRFKNLTASGKVSFLEFFGVPVYQSLAMLRSNNGIMNTLATDGEKKFDFTVQNDGEEVTLKTRSVTAKIAGTLIDEQPLAIYTIDKVLLPKELFKAAAPAPAPEKEVA
SsuFLA21	MKNHFSVFLFSAILLSLHCNQTLSQSPAAAPAKAPAAASAPPPAATSTAQASPPVMVPVQVSKGPVNVIKILQKAGGFAVFIRLIKSTQEDIQVFSQLNDSRDGVTIFAPTDGAFSAIIKSGVLNSLSVLNSLSDHQKIELVQFHIIPKILTTANFQTVSNPITTLAGSGSRFALDVITTENMVNVTSGLTNTSVSAIVYTDSQLAIYQVDKVLLPLDIFAPKSLAPAPSPPKPKKDDGEETPVVPEDISGAVSCAMLNSLVIFGAGMAAAVFPL
SsuFLA22	MLLKLAQSPAAAPGPPGPANVTKILEKGGQFSVFTRLLKATREDVTLNGQLNNTNNAITIFAPSDSAFSSLKPGTLNSLNDQEKAELVQFHIIPQYLSSSQFQTLNVTTAGNSVNITTGLTNTSVSGTIYTDNQLAVYQVDKVLLPVDIFTPKPPPPAPAPEKTRRGSKSAESPDTSVDNSGAVTLNVLDNAVFFGVGIVAVIFSL
SsuFLA23	MKQQCSLSFFSFFLLLLRCTSTFAQSPAAAAPAQAPAAVVAQPPAATPAQAAQPHGAITNVTKILEKAGHFTIFIRLLRSTQEDSHLFSALNDSSSGVTIFAPTDGAFSELKSGTLNTLSDGDKSELVKFHVVPTFLSTSQFQTVTNPLGTWAGTGNRLPLNVTSYPNSVNITTGLTNTSLSGTVYTDSQLAIYKIEKVLLPKDIFASKAPAPAPVAPAPEKPTKAVPAATVESPVAPVDIPVHFCSLKIMWWDPLA
SsuFLA24	MATTPLSLFLLSLLLSLSLYAQAQTPAAPAPTPSGPVNFTAVLEKGGQFVTLIRLLNKTQTLNQIDNQLNSSSEGMTVFAPTDNAFNNLKAGALNDLSQQEQASGPDGVWGLNFTGQSNQVNVSTGLVLVQINNALRQDFPLAVYPVDKVLLPEALFGVKPPSASPPAPSSKSNATEAAAGPTTDNNAAGGRNVALGLVVGLGLVCIGILS
SsuFLA25	MTLHLRTAAPAQAPPAVVAQPPAATPTQAAAPHGITNVTKILEKAGHFTIFIRLLRSTQEENHLFSALNDSSSGLTIFAPTDSAFSELKSGTLNTLSDGDKSELVKFHVVPTFLSTSQFQTVSNPLGTWAGTGSRLPLNVTSYPNSVNITTGLTNTSLSGTVYTDSQLAIYKIEKVLLPKDIFASNAPAPEPVQAPVREKPTKAVPAANVESPVAPVDISSSVTFMQNSVIGSVGIVLAAMFAL

SsuFLA26	MKKQYSISSFSVFILFLHCSNTFAQSPAAAPAQAPPAVVAQPPAATPTQAAAPN<br>GITNVTRILEKAGHFTIFIRLLRSTQEENHLFSALNDSSSGLTIFAPTDSAFSEL<br>KSGTLNTLSDGDKSELVKFHVVPTFLSTSQFQTVSNPLGTWAGTGSRLPLNV<br>TSYPNSVNITTGLTNTSLSGTVYTDNQLAIYKIEKVLLPKDVFGSKAPAPAPA<br>APVREKPTKAVPEATVESPVSPVDISSALMFTHNNVVGSVGMVAAAMFAL
SsuFLA27	MKKQYSISSFSVFILFLHCSNTFAQSPAAAPAQAPPAVVAQPPASTPTQAAAPN<br>GITNVTRILEKAGHFTIFIRLLRSTQEENHLFSALNDSSSGLTIFAPTDSAFSEL<br>KSGTLNTLSDGDKSELVKFHVVPTFLSTSHRLPLNVTSYPNSVNITTGLTNTS<br>LSGTVYTDNQLAIYKIEKVLLPKDVFGSKAPAPAPAAPAREKPTKAATVESPV<br>SPVDISGALMFTHNNVVGSVGIVAAAMFAL
SsuFLA28	MRKQYSLSSFSFFILFLHCTNTFAQSPAAAPAQAPAAVVAQPPAATPTQAAAP<br>HGITNVTKILEKAGHFTIFIRLLRSTQEENHLFSALNDSSSGLTIFAPTDSAFSE<br>LKSGTLNTLSDGDKSELVKFHVVPTFLSTSQFQTVSNPLGTWAGTGSRLPLN<br>VTSYPNSVNITTGLTNTSLSGTVYTDSQLAIYKIEKVLLPKDIFASNAPAPAPV<br>QAPVREKPTKAVPAANVESPVAPVDISSSVTFMQNSVIGSVGIVAAAMFAL
SsuFLA29	MKKQYSISSFSVFILFLHCSNTFAQSPAAAPAQAPPAVVAQPPAATPTQAAAPN<br>GITNVTRILEKAGHFTIFIRLLRSTQEENHLFSALNDSSSGLTIFAPTDSAFSEL<br>KSGTLNTLSDGDKSELVKFHVVPTFLSTSQFQTVSNPLGTWAGTGSRLPLNV<br>TSYPNSVNITTGLTNTSLSGTVYTDNQLAIYKIEKVLLPKDVFGSKAPAPAPA<br>APVREKPTKAVPEATVESPVSPVDISSALIHTSLAHCIVIFFIEH
SsuFLA30	MASCSHWWHAPVYFIASAVLAFIAISTALNSPPSKATRPTRPISNHISLNASRT<br>LRESGFNIMATLLLISPEMFFSSPNTTIFAIKDSSLVNTSIPPWFLKNLLKYHTS<br>PMKLSMEDVFKKPQGSCFPTLVDRKKLAVTKIDAKQRLAEINHVLVSHPDM<br>VLERQIAIHGVLAPFSSLRSKDVYLGWESIQAPICDDNSSLVSDATDPGIVLE<br>WTRIIHLLSSHRFVSFAIGLNSVLDRILADHKNLSSVTIFAPPEFDFVASSSPML<br>ERIVRLHILPQRATYKDLAALPDKHRLRTLLPDVDLEITNDVNVTQGLAING<br>VEIAAPEILSSKEFIVHGITQAFKMAKFPNASR
SsuFLA31	MAALRYSLLLSFTLSALVSTVLAHNITDILSGFPEYSEFNKYLTQTKLADEINT<br>RQTITVLALNNGAMSALAAKHPLSVIKNALSLLVVLDYFDPTKLHQISKGST<br>LSTTLYQTTGNAPGNLGFVNITDLQGGKVGFGSAAPGSKLESSYTKSVKQVP<br>YNISILEISQPIIAPGILTAPAPTPSAGCKTFASLLQTSGVIKMYQSAADKGLTIF<br>APNDEAFKAAGVPDLSKLTNAEVVSLLQYHATASYSPFGSLKTSKDPISTLAS<br>NGAGKFDLTVTSAGDSVTLHTGIGPSRVAETVLDSTPLVIFTVDNVLLPVELF<br>GKAPSPAPAEEPVSAPSPSPIASTPAPAIVEAPSPLAASPPAPPVETPGGAPADA<br>PFGSENSTADGNAAVHVIVPVQVTVFAAVICSILMS
SsuFLA32	MEFSVLIVLSSALLFACAPLAYAQKVASPPAPTPTPSPAPAPSPPYVNLTLLLSV<br>AGPFQNFLNYLESTKVIDTFQNQANNTDEGITIFVPKDDAFKNLKNPSLSNLT<br>QDQLKQLILFHALPHFYSLSDFKNLSQVSPISTFAGAGGYALNFTDSSGTVHL<br>DSGWSKTKVSSSVHATDPVAIYQVDKVLLPEAIFGTDIPPTPAPAPAPETSPTA<br>DSPTSDDTTGSSPANSLPNSSFRINGVGIWSQLVLGIAGVVVLFL
SsuFLA33	MPRPLPLLTLAVSLALLASTATVNAHNITRILAKHPQFSTFNHYLTVTHLAAEI<br>NRRQTITVLALDNAAMSSLLSKQLSVYTLRNVLSLHVLVDYFGTRKLHQITN<br>GTEVTATMFQATGSAPGSSGYVNITDLNGGKVAFGAEDSNGKIDAVYVKSLV<br>EIPYNISVLQISQPLNSAEAEAPTAAPTLNVTAILSKQGCKAFSDLLIASGAHT

	TFEENVDGGLTVFCPTDAVINGFMPKYKNLTAPQKVSLLLYHGIPIYQSLQMLKTSNGLMNTLATNGANKYDFTVQNAGEVVTLETKVTTATITGTVKDEEPLVVYKINKVLLPRELFKAVQKAPAPKGAKVVADGPAADAPSDESVDQTADNNGVNKIDGGRLAVVALSLFYGVVMFS
SsuFLA34	MKRQISFSFSLVLLFLHCSQTLSQPPTAAPAKAPAAATAPPLAATSAQASPPVMVPVQVSKGPVNVIKILQKAGHFAILTRLIKSTQEDIQLFSQLNDSRDGVTIFAPTDGAFSAIIKSGVLNSLTDHQKIELVQFHIIPRVLSTANFQTVSNPITTLAGSGSRFTLDVITTESMVNVTTGLTNTSVSAIVYTDSQLAIYQVDKVLLPLDIFAPKPLAPAPAPPQPKKDDGEESPMIPEDTSGSVTCMVHNTLFMFGVGIVAAAIPL
SsuFLA35	MVPQFLFSASFILFFLLYCPTILAQSPAAAPAPAGPTNVTKVLEKGGQFSVFIRLLKATQEDVTLNGQLNNTNNAITIFAPSDSAFSSLKSGTLNSLNDQEKAELVQFHIIPQFLSSSQFQTVSNPLTTQAGSGGRLELNVTTTGNSVNITTGLTNTSVSGTIYTDNQLAVYQVDKVLLPLDIFTPKPPTPAPAPEKTKKRSKAAASPESPADASGAVSFAVLNNVVFFGVCMVAAIYSL
SsuFLA36	MSLRFSPVFALCFSLLLFFNTARAFNITQILSQYSDFKTFSSYLTETQLAGEINSRQTITVLVVEDGNMSPLSGKPKDEIKNVLSGHVILDYYDVAKLQKLQNKTAMLTTLFQSSGQAVGQQGFLNVTVLSTNSVAFGSAAPGSSLSSNLVKSVSSQPYNISVLQVSNIIVPAGTGKTNSTTSPVTIGSPKTSPTSPASSPEKPPSSSNPPPSKAPAPSTAKPPPTNAPAPSMVKPPAANAPSATPPAMSTSPVEGPSPATADGPSADSPTLSPPAMDGPVAATPAADGPLADAPSDPKSDASVINPGNNLASFAPILLLAFALA
SsuFLA37	MATVLLFFLILLLISSSVLAASSPFSNALEILSASGYLSMALTLQITSKRLNLESSAATIFAPLDIAFARVGQLSALDLQYHISPLRLSGDYLSSLPFGAKIPTLLPNHSLTVTTSLGYFDGKLSINGISIEESALVDFGSLIIFGMGEFFSSSLEISPNLTPAPSPSPVTSLGDTSQNEPSGMDADYLGQASHLLILTIFAPLDQAMEEYAKNASDYSSIFRHHVVPGLFPRQDLEGFNDGTSLPTFFRGFMINLTRSGDVLVLNGVPLIFPDMYQSGWLVIHGLNQLLMPPMKEIVGESFSELDGGEDKPDVLDFDEYVYGSP
SsuFLA38	MDSKVSSLLFIAILFLLSSTSTAFNITKILAQYPEFASFNDLLAQSGLAQEINGRETITVLALDNGSIDGLSGRPVDIAKRILSAHVILDYYDQIKLSKLQKASTIVTTLFQASGIADDRQGFLNVSKTAEGIKFGSAMKGAPLVASLVKSVYAQPYNISVLQVSEPIEAPGIENMAPPPPPAAVPKKAPAPAAKTPSKAPAPSKVEPSTPAESPTEGPVAADVPAASPLADAPLADEVPAKAASSQMHVGGAVVVVGLLACMVGF
SsuFLA39	MAPINPAISHITPTTTLTYFILLLLTTTTTTTPILAITNLTALLSSFPDFSSFTSLIASTPSLTSDLSDRSALTLLVIPNSYLSSSLDLTRRLSPSALADLLRYHILLQYLSFSDLHQVPPSGTLITTLFQTTGRASSNSGSVNTLPYNVSIISVNSLLVPYGFDLMASETRPTLGLNITKELLDGHNFFVAASLLSASGVVQEFEADEGGAGITLFVPTDSAFSDLSATAISLQSLPADKKADVLKFHVLHSYYPLGSLESIVNPVQPTLATEDMGAGSFTLNISRVNGSVAIDSGIVQASVTQTVFDQNPVVIFGVSKVLLPKEIFGRNPVLTSKPGSPDMGNAQPPVSVLSPETSPKMLSSAPGVREEIKSGVGGLQWLSTLPLLFVVFVCNCI
SsuFLA40	MSPQDLMINKSTSKILLHLLLLSLLHQITTAALTDQQLEFALLSLRSHGYTLFPNAISTSDLRFHLLNQTNNATSTFTLFSPPDSLLFSVDLSSTASHYTKSLFLHVS

	PSRLSMSDFRNLTAASGGTYIDSLVPNHRLLINNSQARLNGTVDGSVLVNRVRVSVPDLFLGSDIAVHGLDGILVAGFDDKVEDTSFEAATSSPANEIWSAELNSPPAVRFPARKRNGKNRRRNGKNRRRNGRNGGITRSNHRGRRIHDGFGRGGGRNIGGGTRGDGTHGAFSKYNNRL
SsuFLA41	METFTTLLVLLMKVLVCATSPTGIPSKSQDLVVATNEMARANYFSFVLLINMSPLDQRLQENVTFLMPKDRMLSKIRMHQNAVSGFLLRHSIPSPLLFDHLQHIPPGSLIPSSDPDYMLNISNKGRKNFSLNNVRITSPNLCTAGSSIRCHGIDGVLFVATDRPHLPACSNSSSPAVAPPLPLSDIPPSFPSPAPAGAAAPTDQEHSPKHSGSSHLESPSPGGLLKFVATSILVLNVWVL
SsuFLA42	MATSPLSLLILSLFLSLSLHAQAQAPAAPAPASSGPVNFTAVLEKGGQFVTFMRLLNKTQTFNQIENQLNSSSEGMTIFAPTDNAFNNLKAGALNGLSQQQQVQLLQYHMLSKFYSLSNLLLVSNPVSTQASGHEGVWGLNFTGQSNQVNVSTGLVEVQINNPLRQDFPLAVYPIDKVLLPEELFGVNRTIASPPPPATSSGKSNSSETAAAEPSPGKNSAGGRNVALGSIVGLGLVCMGIIS
SsuFLA43	MKPQYFLSSFSIFLLFLHCTNTFAQSPAAAPAQPPAVVASPPAATPTQAAAPHGITNVTKILEKAGHFTIFIRLLRSTQEENHLFSALNDSSTGLTIFAPTDSAFSELKSGTLNTLSDGDKSELVKFHVVPTFLSTSQFQTVSNPLGTWAGTGNRLPLNVTSYPNSVNITTGLTNTSLSGAVYTDNQLAIYKIEKVLLPKDIFAPNAPAPAPVKPAPEKPAKEVPEVTAESPAASVDISSARIFTRNLVVGSAGLLASAMFSL
SsuFLA44	MKQQLISSFSMFILFLHCANTFAQIPAAAPAQAPAVVVASPPAATPTQAAAPHGITNVTKILEKAGHFTIFIRLLRSTQEENHLFSALNDSSTGLTIFAPTDSAFSELKSGTLNTLSDGDKSELVKFHVVPTFLSTSQFQTVSNPLGTWAGTGNRLPLNVTSYPNSVNITTGLTNTSLSGAVYTDNQLAIYKIEKVLLPKDIFASNAPAPAPVAPAPEKPAKAVPAATVESPAASVDISSALIFTHNLVVGSAGLLASAIFSL
SsuFLA45	MKPQYFLSSFSIFLLFLHCTNTFAQSPAAAPAQPPAVVASPPAATPTQAAAPHGITNVTKILEKAGHFTIFIRLLRSTQEENHLFSALNDSSTGLTIFAPTDSAFSELKSGTLNTLSDGDKSELVKFHVVPTFLSTSQFQTVSNPLGTWAGTGNRLPLNVTSYPNSVNITTGLTNTSLSGAVYTDNQLAIYKIEKVLLPKDIFAPNAPAPAPVKPAPEKPAKEVPEVTAESPAASVDISSARIFTRNLVVGSAGLLASAMFSL
SsuFLA46	MDRLQHLLISLYLLILFINLTKTTAQSPAPAPAPPGPTNVIKILKKAGHFKNFIRLLKSTQLDSNLNSQLGNTNNGLTIFAPSDTAFSVLKTGTLPSLTDQEKLELMQFHIVPMFISSSQFETVSSPLKTHAGSGARTHQHHYFDTVYMDTNLAIYQVDKVLLPLDIFTPKPAPAPAPELKAESESPDDAVSKKDISSGVSFAMLRDTVLFIAATVAAISFSL

Green: Signal peptide; Red:AGP-like glycosylated regions;
Yellow: Fasciclin domain; Gray: GPI-anchor protein;
