# Supplementary material for: Genome-Wide Comparative Analysis of the Fasciclin-like Arabinogalactan Proteins (FLAs) in Salicacea and Identification of Secondary Tissue Development-Related Genes
Source: Int J Mol Sci. 2023 Jan 12;24(2):1481. doi: 10.3390/ijms24021481 (PMC9862198; doi:10.3390/ijms24021481)
Supplement: Supplementary file 1 [file ijms-24-01481-s001.zip › Supplementary Figure S1.pdf]

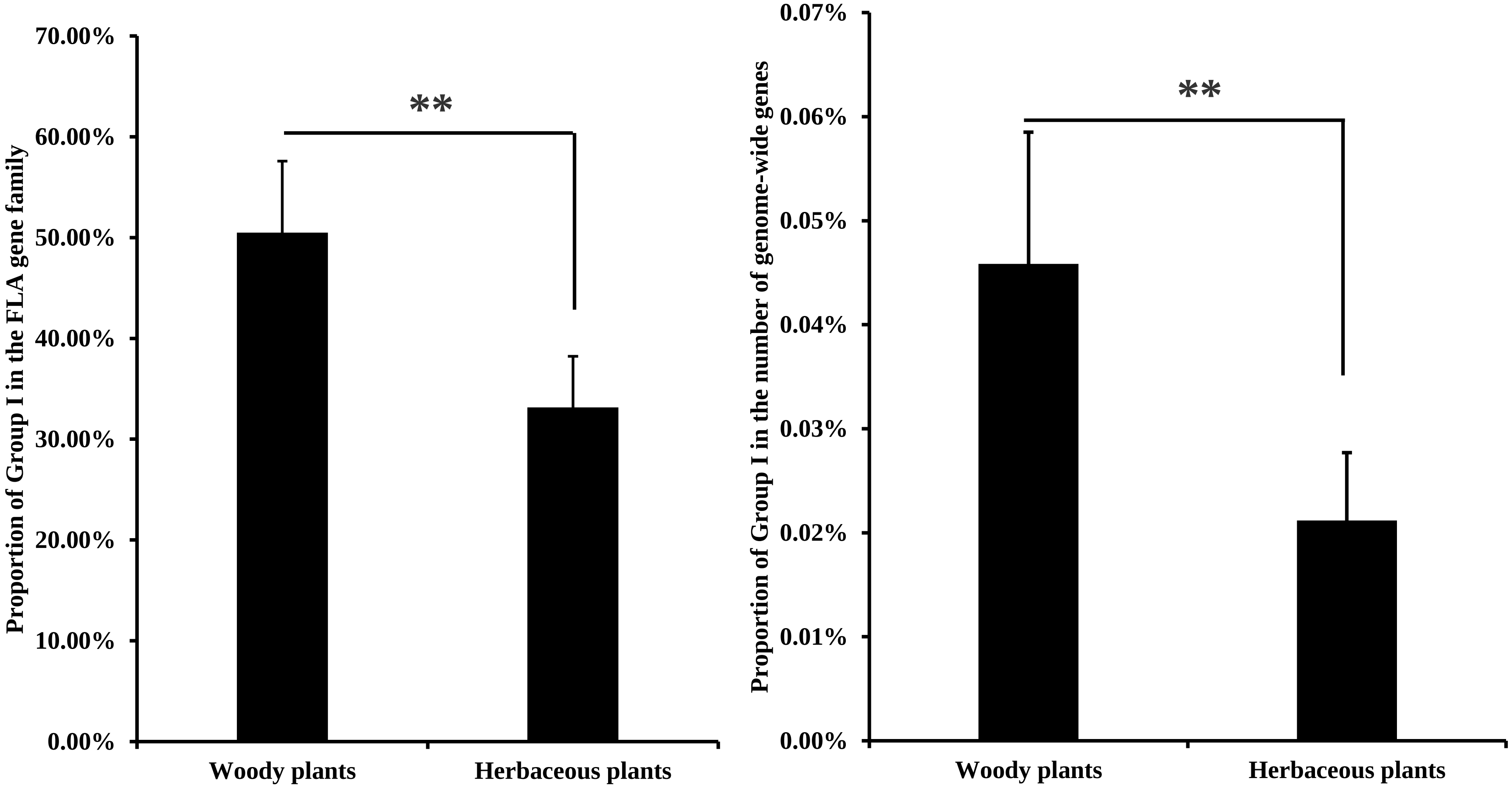

Supplementary Figure S1. Significant ANOVA for the proportion of Group I gene occupancy in woody and herbaceous plants. (A) Proportion of Group I genes in the FLA gene family. (B) Proportion of Group I genes at the genome-wide level. Values shown are the means ± SD of the five woody and herbaceous occupancies, respectively (**$P < 0.01$, two-sided Student's t test). The raw data for statistical analysis are in Supplemental Table S8.
