# Supplementary material for: Genome-Wide Comparative Analysis of the Fasciclin-like Arabinogalactan Proteins (FLAs) in Salicacea and Identification of Secondary Tissue Development-Related Genes
Source: Int J Mol Sci. 2023 Jan 12;24(2):1481. doi: 10.3390/ijms24021481 (PMC9862198; doi:10.3390/ijms24021481)
Supplement: Supplementary file 1 [file ijms-24-01481-s001.zip › Supplementary Materials.pdf]

# Supplementary Material

**Supplemental Table S1.** Prediction of conserved structural domains of FLA proteins in *P. deltoids.*

**Supplemental Table S2.** Prediction of conserved structural domains of FLA proteins in *Salix suchowensis.*

**Supplementary Table S3**. List of the C and N-terminal signals of *P. deltoides* genes identified by BIG-PI Plant Predictor and SignalP online programs.

**Supplementary Table S4.** Prediction of C-terminal GPI anchor attachment sites and N-terminal signal peptides in *S. suchowensis* genes identified by BIG-PI Plant Predictor and SignalP online programs, respectively.

**Supplemental Table S5**. Orthologous genes between *P. deltoides* and *S. suchowensis*.

**Supplemental Table S6.** Ka, Ks and Ka/Ks values of tandem and segmental duplication gene pairs in *P. deltoides* and *S. suchowensis*.

**Supplementary Table S7.** Conserved motifs predicted by MEME program in PdeFLA and SsuFLA.

**Supplemental Table S8.** The proportion of the number of Group I genes in the *FLA* gene family and at the genome-wide level in different species.

**Supplementary Table S9.** Corresponds to the list of p-values in Figure 6D. That is, the list of p-values corresponding to two-sided Student's t-test of relative gene expression between female pollinated and unpollinated treatments for *S. suchowensis* FLA Group I genes.

**Supplementary Table S10.** List of primer sequences used in qRT-PCR reactions.

**Supplementary Sequence S1.** List of the sequences of FLA proteins from eight species: *Corylus avellana L.* (23 members), *Quercus rubra L.* (29 members), *Cinnamomum kanehirae* (23 members), *Cinnamomum kanehirae* (25 members), *Zea mays L.* (26 members), *Cucumis sativus L.* (17 members), *Oryza sativa L.* (27 members,) and *Arabidopsis thaliana* (21 members).

**Supplementary Figure S1.** Significant ANOVA results on the proportions of Group I genes in woody and herbaceous plants.
